# Thermoresponsive Ionic Liquid/Water Mixtures: From Nanostructuring to Phase Separation

**DOI:** 10.3390/molecules27051647

**Published:** 2022-03-02

**Authors:** Nancy C. Forero-Martinez, Robinson Cortes-Huerto, Antonio Benedetto, Pietro Ballone

**Affiliations:** 1 Institut für Physik, Johannes Gutenberg-Universität Mainz, Staudingerweg 9, 55128 Mainz, Germany; forero@mpip-mainz.mpg.de; 2 Max-Planck Institute for Polymer Research, Ackermannweg 10, 55128 Mainz, Germany; 3 School of Physics, University College Dublin, 94568 Dublin, Ireland; antonio.benedetto@ucd.ie (A.B.); pietro.ballone@ucd.ie (P.B.); 4 Conway Institute for Biomolecular and Biomedical Research, University College Dublin, 94568 Dublin, Ireland; 5 Department of Sciences, University of Roma Tre, 00146 Rome, Italy

**Keywords:** thermoresponsive solutions, ionic liquids, UCST, LCST, nanostructured liquids, computer simulation, desalination, forward osmosis

## Abstract

The thermodynamics, structures, and applications of thermoresponsive systems, consisting primarily of water solutions of organic salts, are reviewed. The focus is on organic salts of low melting temperatures, belonging to the ionic liquid (IL) family. The thermo-responsiveness is represented by a temperature driven transition between a homogeneous liquid state and a biphasic state, comprising an IL-rich phase and a solvent-rich phase, divided by a relatively sharp interface. Demixing occurs either with decreasing temperatures, developing from an upper critical solution temperature (UCST), or, less often, with increasing temperatures, arising from a lower critical solution temperature (LCST). In the former case, the enthalpy and entropy of mixing are both positive, and enthalpy prevails at low *T*. In the latter case, the enthalpy and entropy of mixing are both negative, and entropy drives the demixing with increasing *T*. Experiments and computer simulations highlight the contiguity of these phase separations with the nanoscale inhomogeneity (nanostructuring), displayed by several ILs and IL solutions. Current applications in extraction, separation, and catalysis are briefly reviewed. Moreover, future applications in forward osmosis desalination, low-enthalpy thermal storage, and water harvesting from the atmosphere are discussed in more detail.

## 1. Introduction

Thermoresponsive systems are “a special case of” responsive materials [1,2], whose properties crucially depend on external stimuli, which, in the thermoresponsive case [3], primarily consist of a change in temperature. Since every real material will react to changing conditions, such as temperature and pressure, the important role in the definition is played by the *crucial* qualification of the change displayed by the system. In the present discussion, the systems of interest are solutions of organic salts in water and, to a lesser extent, in other solvents. Then, the crucial response to changing temperature will be a reversible transition between a homogeneous state and a phase-separated one, consisting of salt-rich and solvent-rich phases separated by a relatively sharp interface. Moreover, organic salt belongs to the room temperature ionic liquid (IL) variety, which, in recent decades, has been the subject of chemical physics research studies and there are high expectations for its use in advanced applications [4]. Since the major portion of this discussion will concern water solutions, the temperatures of interest will be primarily the 0≤T≤100 °C range of liquid water. Despite these drastic limitations, the topic still covers a broad range of systems, phenomena, and applications.

**Figure 1:**
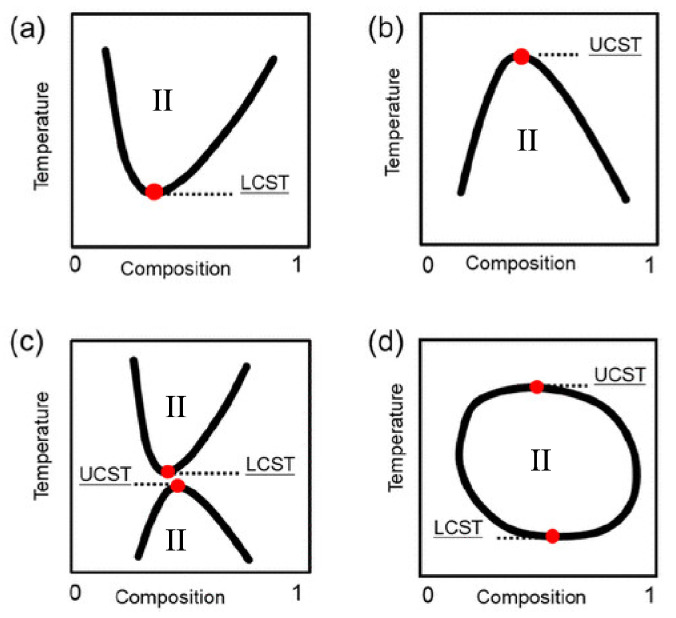
Topology of phase diagrams showing UCST and LCST states (red dots). The full line separates homogeneous and biphasic (II) states. Slightly adapted and reprinted with permission from reference [6].

The solubility gap—as a function of temperature that defines a thermoresponsive fluid—may manifest itself in two major ways (see Figure 1). In most cases, the system will be mixed at high temperatures and demixed at low temperatures, having a so-called upper critical solution temperature (UCST) (see reference [5] in the IL context). In a smaller number of cases, displaying a so-called lower critical solution temperature (LCST), the system is mixed at low temperatures and demixed at high temperatures, a somewhat counterintuitive behaviour, given the role of positional (ideal) entropy.

A solubility gap will result in stable biphasic systems, which are widely used as heterogeneous reaction media, but especially for separation and extraction [7] based on the directional diffusion of organic and biological molecules or metal ions across the liquid–liquid interface. In a genuine biphasic system, chemical species travel macroscopic distances to reach the interface. A thermoresponsive transition provides a way to transform a homogeneous state—in which reacting species are in close contact—into a biphasic state, in which species are segregated, having been separated through a collective mechanism not necessarily diffusion-limited. Since it is based on an equilibrium property, the mixing/demixing transition is reversible, which is an essential feature for several applications.

Needless to say, a solubility gap as a function of temperature is not an exclusive property of IL solutions. Even before the quest for smart materials, several studies have analysed the stability with respect to demixing of homogeneous solutions made of solute molecules dissolved in water or other solvents [8]. Examples of neutral mixtures displaying an upper critical solution temperature are easy to find [8,9,10]; moreover, several cases of LCST are documented in the literature [11]. The complexity of the phase behavior of even simple fluids is illustrated by the systematic (although not fully exhaustive) discussion of the phase diagrams derived from the van der Waals equation of state for binary fluid mixtures (therefore a strictly limited selection) reported in reference [12]. Another related research field has been (and still is) represented by thermoresponsive systems made of polymers dissolved in molecular solvents [13,14,15]. In the language of polymer physics, the homogeneous state corresponds to good solvent conditions, characterised by extended chains, while the demixed state corresponds to poor solvent conditions and collapsed chains. Therefore, in UCST cases, chains are collapsed below $T_{c}$ and extended above $T_{c}$. The reverse occurs for LCST systems. Regarding polymer, most thermoresponsive polymer/water solutions display LCST, while UCST systems are harder to find. The opposite seems to be true for polymers dissolved in organic solvents [16].

In the present review, the focus is on IL/solvent systems, whose solute, i.e., the ILs, have intermediate complexity between simple fluids and polymer solutions, although the upper limit of solvent and especially solute complexity is left unspecified. Present and conceivable future applications greatly expand the scope of the discussion, covering separation and purification technologies [17] biophysics, desalination, and low enthalpy heat storage. These technologies are increasingly important, for instance, in the environmental context, to extract metal pollutants from water, or to increase the future availability of fresh water. The many applications in catalysis are only briefly mentioned since a specific and more chemical oriented review is available in reference [18] (see also reference [19] for catalysis in thermoresponsive non-IL solutions).

The first major conceptual point in analysing thermoresponsive liquid mixtures concerns the nature of the critical point that gives UCST and LCST systems their name. Mixing/demixing of neutral fluid mixtures belongs to the 3D Ising universality class [20,21]. The dynamics of the transition, investigated again for neutral species, is also heavily affected by these universality properties [22]. Of course, the presence of mobile electrostatic charges, and long range Coulomb interactions need to be carefully taken into account.

The simplest model of salt in a solution is provided by the so-called restricted primitive model (RPM), consisting of hard spheres of equal diameters and opposite charges moving in a frictionless dielectric continuum, which plays the role of an implicit solvent. In this idealised picture, the liquid–vapour coexistence curve predicted by theories and determined by the Gibbs-ensemble Monte Carlo simulation [23] is interpreted as a solubility gap between a salt-rich (the liquid) and a solvent-rich (the vapour) phase, at moderate temperatures, terminating at a critical point representing the UCST of the solution. The criticality class of this UCST was long-believed to be the mean field, as suggested by the long range of potential [24]. However, screening affects the range of the effective interactions, and the present consensus is that even for RPM, the criticality class is 3D Ising, having critical exponents β=∼0.325, γ∼1.24, ν∼0.63. In the context of our discussion, the UCST of RPM is important, since it shows that Coulomb interactions alone are sufficient to drive phase separation. Moreover, if the dielectric constant of the implicit solvent is assumed to be a decreasing function of *T*, the upper critical point might turn into a lower critical point, showing a way in which LCST or even closed-loop miscibility gaps might arise [25].

The RPM, however, is far from being an adequate model for ILs, which, even in the pure bulk phase differ from a primarily Coulomb paradigm in many essential ways [26], exemplified by their low melting temperature, large deviation of electric conductivity from the Nernst–Einstein relation, or, in water solution, by a relatively low osmolality with respect to a fully dissociated electrolyte. In IL/water solutions, therefore, the liquid–liquid solubility gap may arise from specific ion–water interactions, due to dispersion energy or related to hydrogen bonding (HB) or lack thereof [27]. In the literature, these cases are referred to as solvophobic phase separation [24]. The effect of hydrogen bonding on solubility/miscibility is also apparent in solutions of IL with several organic solvents. Moreover, in a few cases, hydrogen bonding may occur between anion pairs (anti-electrostatic hydrogen bonding [28]), promoting ion aggregation and demixing. Despite the variety of microscopic interactions and mechanisms, the phase separation in solutions of ILs and molecular solvents still belong to the 3D Ising universality class, as summarised in the following paragraph.

**Figure 2:**
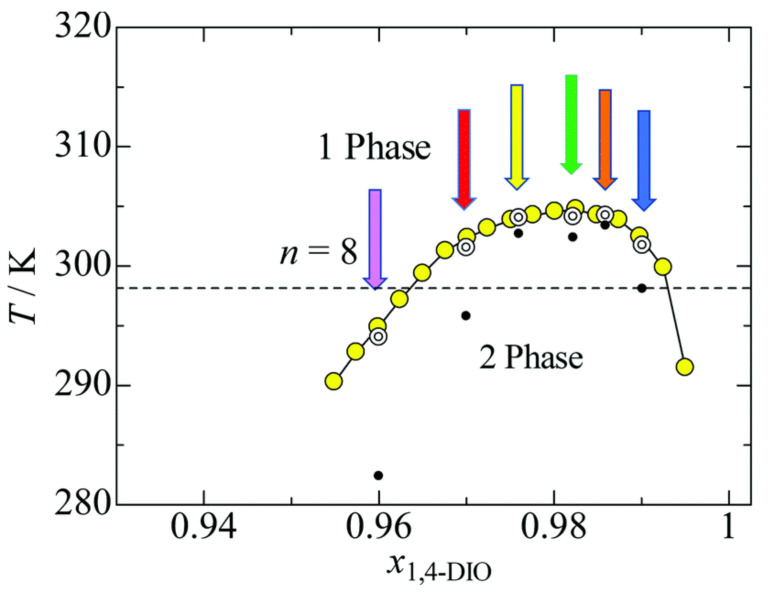
Characterisation of the UCST line in [C${}_{8}$mim][NTf${}_{2}$] in 1,4-dioxane. $x_{1,4-DIO}$ is the molar concentration of dioxane in the solution. The arrows show the direction in the ($x_{1,4-DIO},T$) plane followed by measurements to characterise the system critical properties. Reprinted with permission from reference [32].

A detailed and quantitative early analysis of critical properties of an IL/water solution is reported in reference [29], with the IL being choline bis(trifluoromethylsulfonyl) [Chol][NTf${}_{2}$] (see also reference [30], on [bmim][BF${}_{4}$], and reference [31] on [N${}_{3444}$][I]). These studies find 3D Ising critical exponents, although such a behaviour might only be observed in the close vicinity of the critical point situated at concentration $x_{c}$ and temperature $T_{c}$. The early interest in critical point properties of simple and complex electrolytes has not been retained in more recent discussions. The exception to this statement is the very recent study of criticality for the UCST of 1,4-dioxane solutions in the [C${}_{8}$mim][NTf${}_{2}$] IL [32]. Once again, close to the critical composition and temperature, 3D Ising critical exponents are found. However, moving away from the critical composition, correlation length and optical thickness measured at constant concentration and approaching the spinodal temperature from above (i.e., in the homogeneous phase, see Figure 2) give mean field exponents. Away from $x_{c}$, however, these are not genuine critical exponents; therefore, the result is in fact compatible with the most recent accepted picture of 3D Ising exponents at criticality. Admittedly, up until now, the quantitative determination of critical properties of IL solutions has not been extensive, having been limited to only a few thermoresponsive cases. However, critical phenomena are the realm of universality [33], and the few explicit results that are available are sufficient to state that the critical exponents for the demixing of IL solutions at UCST and LCST are 3D Ising.

Anticipating the broad picture that emerges from experiments and computational studies, one can say that UCST and LCST are equilibrium transitions driven by enthalpy and entropy, respectively. Moreover, since hydrophobicity/hydrophilicity play a role, the number and structure of hydrogen bonds are important quantitative aspects, and, as a matter of fact, they depend mainly on the nature of the anion. The entropy variations that underlie the peculiar demixing with increasing temperature in LCST systems are due primarily to the hydration/dehydration of the ions: hydration, especially when due to strong and highly directional hydrogen bonds, decreases the entropy of the system since it reduces the reciprocal freedom of water and ions. Hence, dehydration, which is an integral part of the phase separation, is accompanied by a surge in entropy. Another way to look at LCST is to conjecture the formation of hydrates at low temperature [34].

A remarkable phenomenon occurring in ILs is nanostructuring [35], observed for pure IL and IL/water (or, more generally, IL/solvent) mixtures [36]. In the case of IL/water solutions, nanostructuring is observed for amphiphilic IL compounds. The decrease of the free energy of water/IL interfaces together with the tendency to segregate hydrophobic domains give origin to nanometric aggregates in a system that retains its overall homogeneity. The amphiphilic character of IL giving origin to nanostructuring, and the moderate hydrophobicity of IL underlying thermo-responsiveness are not necessarily the same property, but they are clearly related aspects. More importantly, our computational investigations of thermoresponsive IL/water mixtures show a contiguity between nanostructuring and phase separation. This point of view is supported by several previous experimental and computational investigations, as will be discussed in the following sections.

Since this paper is not meant to be a general review on ILs, whose properties however represent the necessary background for our discussion, we point the reader to a series of reviews (see the list in reference [37]) published in the *Chemical Review* (ACS) special issue, and recent reviews on industrial and environmental applications of ILs, references [38,39,40]. An additional important aspect that is relevant (in view of applications) but is only superficially mentioned in the review, concerns toxicity and adverse environmental impacts of ILs. For reasons of length, we only report the most conventional point of view, noting that several ILs are only moderately or not toxic, and their low volatility limits their dispersion in the environment. The full picture, however, is far more complex, as discussed in the recent review in reference [41].

We add here a few considerations on notation. Alkyl-phosphonium and -ammonium cations are the most common constituents of the ILs relevant for this review. They will be denoted by [P${}_{ijkl}$]${}^{+}$ and [N${}_{ijkl}$]${}^{+}$, respectively, with *i*, *j*, *k*, and *l* being the number of carbon atoms in the alkyl chains. Popular alkyl substituted methylimidazolium cations will be denoted by [C${}_{i}$mim]${}^{+}$, where *i* is again the length of the alkyl chain expressed in carbon atoms. Short chain members of this family have a conventional name, i.e., [dmim]${}^{+}$ for i=1, [emim]${}^{+}$ for i=2, [pmim]${}^{+}$ for i=3, [bmim]${}^{+}$ for i=4. The choline cation will be denoted by [Chol]${}^{+}$. Anions that will be referred to without their systematic chemical name will be bis(trifluoromethylsulfonyl)imide (bistriflimide, [NTf${}_{2}$]${}^{-}$), trifluoromethanesulfonate (triflate, [TfO]${}^{-}$) and trifluoroacetate ([TFA]${}^{-}$). A short series of benzenesulfonates ([BnzSO${}_{3}$]${}^{-}$) homologous species will be considered as well: toluenesulfonate [TsO]${}^{-}$, 2,4-dimethylbenzene sulfonate [DMBS]${}^{-}$, 2,4,6-trimethylbenzene sulfonate [TMBS]${}^{-}$ Anions derived from amino acids (AA) will be indicated with the corresponding three-letter abbreviation such as [Ala]${}^{-}$, [Cys]${}^{-}$, etc.

## 2. Theoretical, Computational, and Experimental Methods

The presence of a thermally activated mixing/demixing transition in the phase diagram of a binary liquid mixture is a genuine thermal equilibrium feature, whose first analysis, therefore, relies on thermodynamics.

Starting from the demixed state, the system upon mixing will change its enthalpy and entropy by $\Delta H_{mix}$ and $\Delta S_{mix}$, respectively. At given *T* and *P*, the variation of Gibbs free energy upon mixing is:

$$\Delta G_{mix}=\Delta H_{mix}-T\Delta S_{mix}$$

and the system will spontaneously sit on the side of lowest *G*. If $\Delta H_{mix}>0$ and $\Delta S_{mix}<0$, the biphasic state is stable at all *T*. Similarly, if $\Delta H_{mix}<0$ and $\Delta S_{mix}>0$, $\Delta G_{mix}$ is always negative, and the system is mixed at all *T*. Thermoresponsive systems have $\Delta H_{mix}$ and $\Delta S_{mix}$ of the same sign. The enthalpy term will prevail at low *T*, and the reverse will be true at high *T*. Hence, when $\Delta H_{mix}<0$ and $\Delta S_{mix}<0$, the system is mixed at low *T*, will demix with increasing *T*, and present a LCST. When $\Delta H_{mix}>0$, and $\Delta S_{mix}>0$, the system presents a UCST, being demixed at low *T*, and mixed at high *T*. The $\Delta H_{mix}$ and $\Delta S_{mix}$ will depend on concentration; therefore, the transition temperature will also be concentration dependent (see Figure 1).

Therefore, the first definite statement is that, in UCST systems, demixing is due to enthalpy, while in LCST systems, it is driven by entropy. In IL/water solutions, the latter case is less common than the former, and also somewhat counterintuitive, which makes the LCST even more intriguing. Both cases underlie important applications, and sometimes the same type of application, such as catalysis [18] or desalination [42,43], can be implemented using either UCST or LCST systems.

From this qualitative starting point, the quantitative determination of phase equilibria requires models for the thermodynamic properties of the IL/molecular solvent systems. Equilibrium of phases and liquid–liquid equilibrium (LLE), in particular, requires pressure to be constant throughout the system (mechanical equilibrium), and the chemical potential of each species to be the same in the two or more coexisting phases (chemical equilibrium). Analytical or numerical solutions of thermodynamic models enforcing these conditions provides coexistence lines in the (*composition x*, *T*) plane, possibly identifying UCST and LCST states.

As briefly outlined in the following paragraphs, the most microscopic method, in principle able to provide the required thermodynamic information, is molecular dynamics (MD) based on empirical atomistic force fields or relying on an ab-initio (usually density functional) potential energy surface [44]. Both varieties of MD, however, are too time consuming (especially the ab-initio ones) and also too involved to provide the extensive mapping of thermodynamics functions required for a comprehensive thermodynamic investigation of phase equilibria. In practice, MD simulations are used to verify whether at given (P,T,x) conditions a system is homogeneous or biphasic, letting a sample to run until it reaches equilibrium. However, the target of much thermodynamic research on liquid–liquid equilibria consists of screening broad families of compounds for UCST and LCST behaviour, or involves the determination of thermodynamic functions over wide portions of the phase diagram. These applications are necessarily the realm of semi-empirical thermodynamics models. Time-honored examples are provided by the van der Waals model, or the Flory–Huggins used for IL/polymer solutions. More recently, a panoply of models, and even entire families of models have been developed to achieve these aims, differing for the choice of interactions and effects to be included or excluded, for the target accuracy, and for the admissible complexity and cost of the phase diagram determination. The full description and discussion of thermodynamic modelling of IL and IL/solvent systems is beyond the scope of the present review. For completeness, we briefly mention the major approaches, and we refer the reader to recent reviews (see for instance reference [45]), and to a pedagogical account, freely available from the web, covering several of these models (see reference [46]).

Thermodynamic models can be expressed in terms of the excess Gibbs free energy as a function of the total number of particles *N*, pressure *P*, temperature *T* and composition *x*:

(1) $$G^{exc}\left(N,P,T,x\right)=RT\sum_{i}\ln\gamma_{i}$$

where *R* is the ideal gas constant, the sum extends over the chemical species (ions, solvent) *i*, whose activity coefficient is $\gamma_{i}$.

Equivalently, models can provide the equation of state (EOS) f(P,V,T,x)=0 of the multicomponent fluid system. Once the activity coefficient or the equation of state of the mixture has been modelled, minimisation of the Gibbs free energy provides a complete map of the phase diagram. As already mentioned in the introduction, even simple models, such as van der Waals for binary mixtures, provide a bewildering variety of phase diagram topologies, including UCST, LCST, and loops with both UCST and LCST states [12,47].

Activity coefficient models include the non-random two-liquid models (NRTL) [48,49], the universal quasi chemical activity coefficient model, UNIQUAC [50], and the universal quasi-chemical function group activity coefficient, UNIFAC [51]. Directly or indirectly, these models rely on the Wilson theory for the local composition [52], which, as the name suggests, accounts for correlations in the density of different species, deviating from the statistical average because of specific interactions. This approach, in particular, introduces the effect of association among molecules, due to hydrogen bonding. The NRTL approach accounts only for enthalpy contributions expressed as a function of the local coordination of all species, while UNIQUAC and UNIFAC introduce additional entropy terms. These two last models differ in their strategy to parameterize these excess contributions. UNIQUAC, in particular, is based on the parameterization of contributions from whole molecules and ions, while UNIFAC subdivides species into functional groups before carrying out the parametrization. The strategy of building models by adding contributions from the functional groups in a linear or correlated way, is in fact a general approach (group contribution models) used extensively to predict a wide variety of properties, looking for regularities and correlations among vast numbers of chemical compounds.

Equation of state models often rely on a polynomial expansion for the factor Z=PV/RT in powers of the molar volume $V_{m}$ of the different species. The lowest order able to predict the equilibrium of phases terminating into a UCST or LCST is the cubic one [45], and the simplest prototype of cubic EOS is in fact the van der Waals EOS. Present days versions of EOS models include the Redlich–Kwong [53,54], Peng–Robinson [55], Patel–Teja [56].

All of these methods, relatively successful for simple molecular fluids, are unable to provide a satisfactory model for complex molecular units, including most ILs. A significant improvement is provided by statistical associating fluid theories (SAFT) [46,57,58], which are based on free energy perturbation theory with respect to simpler statistical mechanics model of known free energy. For instance, variable range potential SAFT-VR relies on the known properties of the hard sphere fluid, and PC-SAFT is based on the properties of hard-sphere chains. In the case of ionic liquids, a variety of contributions due to polarity, Debye–Hückel screening, etc., are added.

All of the models briefly outlined, until now, have required an initial challenging parametrization, whose design and quality determine the success of the module. In this respect, these methods are not really predictive, but they primarily represent interpolations over known ranges of structure, interactions and conditions. To describe mixing/demixing, in particular, they need at least one parameter characterising the cross interaction of the two components, often represented by the solubility or activity $\gamma^{\infty }$ of one species into the other at infinite dilution.

**Figure 3:**
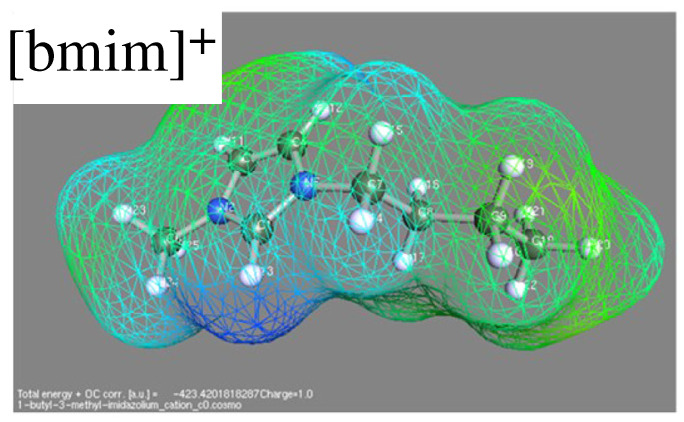
Geometric surface representing the boundary between the conducting or dielectric continuum and an embedded [bmim]${}^{+}$ cation in a COSMO-RS parametrization stage. Reprinted with permission from reference [62].

Predictive models are represented by COSMO-like models, where COSMO stays for conductor-like screening model [59,60]. In principle, this approach requires in input the molecular structure only, which is refined by quantum chemistry computations for the molecule embedded into a conducting (the original and several present versions) or dielectric cavity (a few recent versions of the model, see for instance reference [61]) whose shape closely follows the molecule geometry (see Figure 3). The distribution of screening charge on this separation surface provides the descriptor for the estimation of properties. In many ways, it is a the precursors of machine learning. Application of COSMO to ILs and their water solutions requires the addition of long range electrostatic, sometimes modelled by Pitzer–Debye–Hückel. The results of the model depend somewhat on the ab-initio approach (including the completeness of the basis set) used to determine the surface charge. This dependence, however, does not seem to be very systematic [60], since improving the ab-initio part does not necessarily improve the model predictions. The results depend also on the approach used to describe long range Coulomb interactions and screening in the system.

A secondary role in the thermodynamic modelling of UCST and LCST systems, and also in the polarizable and/or coarse grained models briefly discussed below, is played by the dielectric constant of the IL and of water [61]. Because of the non-vanishing DC conductivity of ILs, however, their dielectric constant cannot be measured by static capacitance measurements [63]. However, the dielectric constant can be estimated as the zero-frequency limit of the dielectric function, determined by dielectric spectroscopy. Moreover, this route is not free of difficulties, because electrode polarisation effects prevent a precise measurement of ϵ(ω) over a broad range of low frequencies. As a result, values of the dielectric constant are available for a fairly high of ionic liquids, but are affected by non negligible uncertainties (∼5−10%). The available values span a wide range [63], from ϵ≊15 up to ϵ≊100.

The thermodynamic models briefly outlined in this section are very general, and extensively used to predict a large number of properties of ILs and their solutions. Their application to LLE, and to UCST, LCST in particular, has been far less extensive, but sufficient to formulate a few recommendations, as reported in the following Section 3.

Any of the models listed above for the Gibbs free energy as a function of (T,P) and composition is also the first major ingredient of a classical density functional theory of fluid mixtures [64]. Assuming, for the sake of simplicity, a local density or gradient expansion approximation for the volume density of excess free energy g(r|T,P,x) at position r, one easily obtains a model for the system free energy G(T,P,x) that allows to investigate the structure and stability of the interface between two phases of different composition [65]. This, in turn, represents an alternative route to determine phase equilibria, since a biphasic state requires a stable interface between the two phases, whose destabilisation with changing thermodynamic conditions marks the proximity of the mixing/demixing transition. These simple considerations outline a seldom discussed relation between different research subjects, whose exploration could benefit both the thermodynamic modelling of phase equilibria and classical density functional theory.

Recently, machine learning has been added to the panoply of approaches devised to predict IL properties [66] including their equation of state and LLE. An intermediate approach between traditional thermodynamic models and machine learning arguably is represented by structure-property quantitative relations, SPQR [67] (also known as quantitative structure–property relations, QSPR). These approaches apply multivariate analysis to model and then predict properties starting from geometric parameters (descriptors) derived from the structure of molecules or ions in the system. Once trained on a suitable database, they hold the promise to predict virtually every system property, including the UCST and LCST of solutions. The challenge, however, is precisely the choice of the training set, which should adequately represent the chemical space of interest. In this respect, the vast number and variety of ILs represents a serious challenge, and even more problematic is to ensure that all the data in the training set are compatible and consistent, a condition that has been questioned in very recent papers [68] However, given the practical interest in the virtual screening of compounds and the power of present computational resources, there is little doubt that methods of this kind will acquire a growing role. In the meantime, the formal matching of machine learning and QSPR has been proposed in reference [69,70]. The resulting algorithm, however, has not been applied yet to UCST and LCST properties of IL solutions.

**Figure 4:**
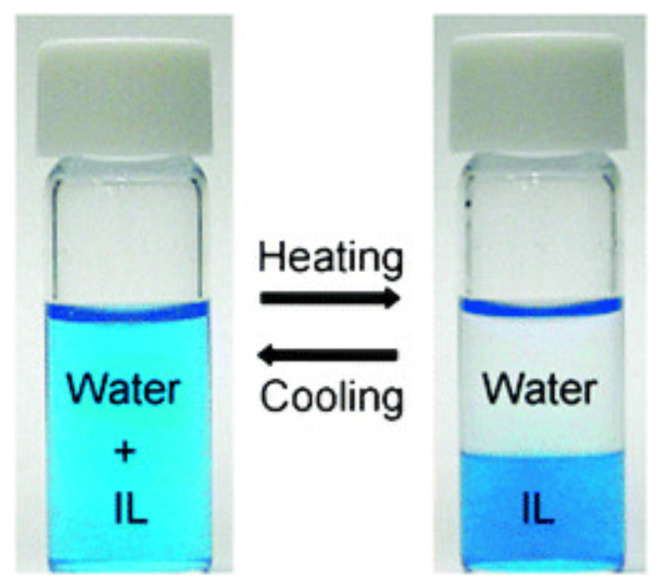
Visual detection of LCST transition in 35 wt% [P${}_{4444}$][CF${}_{3}$OO] solution in water. Cooling and heating are of the order of 5 °C. A blue dye soluble in the IL, but not in water, has been added to identify the volume occupied by the IL. Reprinted with permission from reference [71].

**Figure 5:**
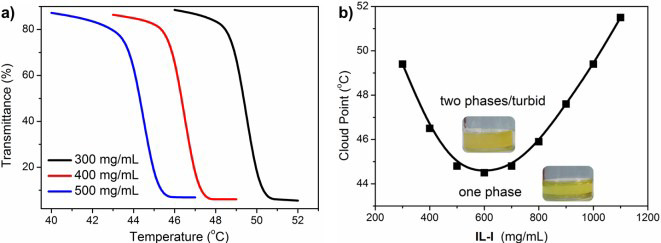
(**a**) light transmittance through a mixture of 1,3-dimethylimidazolium iodide (IL-I) in acetone; (**b**) dependence of the cloud point temperature on composition for the same system, displaying UCST. Reprinted with permission from reference [74].

From the experimental side, several papers on UCST and LCST visualise mixing/demixing showing pictures of liquid samples at temperatures bracketing the transition (see, for instance, Figure 4 [71]). The phase separation, manifesting itself in the stratification of two phases of different density, can be made more easily detectable adding a dye soluble into one phase only in the biphasic system. This macroscopic identification of demixing can be rendered automatic by measuring with a spectrophotometer the temperature and/or concentration dependence of the transmittance of a beam of light through the sample, which shows a drop at the cloud point of the mixture (see Figure 5). In practice, the cloud point is intermediate between the coexistence and the spinodal (stability) lines of the system [72]. The simultaneous measurement of the rotational power of the sample on a linearly polarised light beam allows to detect the formation of chiral liquid crystal phases, even for achiral molecules [73].

Thermodynamic measurements include differential scanning calorimetry to identify phase changes in the pure IL and in their solutions. Moreover, measurements of thermal and electric conductivity, diffusivity (by quasi-elastic neutron scattering) and viscosity contain information on the dynamics of molecules in mixtures approaching criticality from the one-phase side [29]. The formation of nanometric and micrometric structures, again in the homogeneous state approaching the UPST or LCST lines, can be detected by dynamic light scattering (DLS) measuring time correlations in the intensity of light scattered from fluctuating domains in the system, and by neutron (SANS) and X-ray (SAXS) small angle scattering. Other thermodynamic experimental methods are listed and briefly discussed in reference [75].

A variety of other methods target molecular level properties, although averaged over the entire sample. The most powerful technique, providing information on the conformation and bonding of ions and water, is certainly NMR, applied to atomic level probes such as ${}^{1}H$, ${}^{13}$C and ${}^{15}$N. Both 1D and 2D [76] NMR have been used, characterising the bonding environment of the target nuclei and the dynamics of the ions through frequency shifts as well as spin-lattice and spin-spin relaxation times. Additional information on the geometry and distribution of hydrogen bonds is often acquired by X-ray diffraction on crystallised samples [29]. As in every other complex chemical system, vibrational spectroscopy, such as infrared (IR) and Raman, can probe inter- and intra-molecular bonding. Optical spectroscopy, and UV/Vis absorption spectroscopy in particular, is used less often to gain further insight on bonding and on its changes through thermally driven mixing/demixing transitions.

The variety of aims motivating the experimental investigation of thermoresponsive IL/water solutions, from the quantitative determination of critical properties, to the screening of thermo-responsiveness over families of compounds and to practical applications, underlies the variety of methods and the different standards of chemical purity and measurement accuracy adopted in different studies. The most demanding standards have been used in quantitative studies of critical state properties. According to reference [31], on the critical properties of [N${}_{3444}$][I] in water, the solution was prepared from a colourless salt in which no sign of impurity was detected by ${}^{1}$H NMR or by IR spectroscopy. The chemical stability of the salt and of its water solution were tested over one month in nitrogen atmosphere, finding virtually no decomposition. The coexistence curve was determined on samples whose temperature was controlled to better than 0.01 K, and whose composition allowed the determination of the critical composition $x_{c}$ with up to four digits.

Recent explorations of thermo-responsiveness are less strict, declaring a purity of the salt component of 98.5 wt%, used without further processing, with a temperature control approximately one order of magnitude less strict. The requirements are even less stringent in several studies concerning applications, not least because higher standards would be difficult to enforce outside the lab.

**Figure 6:**
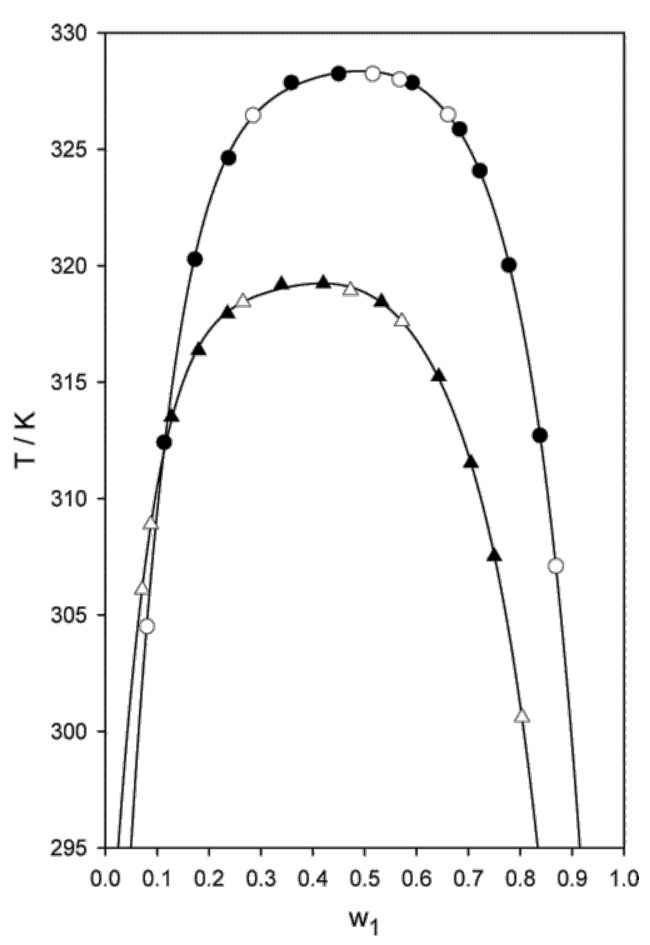
LLE coexistence curves for [bmim][NTf${}_{2}$] in cyclohexanol (triangles) 1,2-hexanediol (circles) as a function of mass fraction $w_{1}$. Filled symbols represent samples whose water content is 160±30 ppm; empty symbols represent samples whose water content is 480±50 ppm. Reprinted with permission from reference [77].

No systematic study is available in the literature of the effect of impurities (halides, metals, water, products of thermal or chemical decomposition) on UCST and LCST, but a rough estimate of the role they could play can be gained from the results for the coexistence curve of ILs dissolved in non-water solvents, in which water plays the role of an impurity. The results of reference [77], comparing the coexistence line of [emim][NTf${}_{2}$] in three alcohols (propan-1-ol, butan-1-ol, and pentan-1-ol) contaminated by two different water concentrations (160±30 ppm and 480±50 ppm) are encouraging, since for each nominal system the two curves for lower and higher water content are nearly indistinguishable (see Figure 6). The insensitivity of the coexistence curve on impurities very likely does not extend to other properties, and especially to dynamical properties, such as diffusion, thermal, and electric conductivity, viscosity, which are know to be affected by contaminants and by water in particular. These properties, however, have been seldom discussed in experimental papers concerning UCST and LCST of Ils in water or other simple solvents.

Perhaps because the major focus of experimental papers is on the coexistence curve and on the (*x*, *T*) location of UCST and LCST, the results reported and discussed by various papers on the subject appear to be rather consistent, despite the different and sometimes not outstanding standards.

A different issue for the accuracy and reproducibility of results concerns the chemical stability of all compounds over a ∼100 °C temperature interval, and especially upon mixing ILs with solvents. This, however, will be briefly discussed in the next section.

Neutron and X-ray scattering represent two of the major experimental tools to probe the structure of liquid mixtures on several different length scales [78]. The most detailed information that can be extracted from the scattered intensity is represented by the partial structure factors. In a homogeneous and isotropic system, the k≠0 components of the density operators:

(2) $$\rho_{\alpha }\left(k,t\right)=\sum_{j\in \alpha }\exp\left[ik\cdot r_{j}\left(t\right)\right]$$

(k=∣k∣) represent fluctuations at time *t* in the distribution of scattering centres of type α, whose positions are $r_{j}\left(t\right)$; in this expression, k is a wave vector. Then, the partial structure factors:

(3) $$S_{\alpha ,\beta }\left(k\right)=\frac{1}{N}{\langle \rho_{\alpha }\left(k,t\right)\rho_{\beta }\left(-k,t\right)\rangle }_{t}$$

measure correlations in the fluctuations of species (α, β). In this equation, *N* is the total number of scattering centres in the system, and ${\langle \ldots \rangle }_{t}$ means average over time.

In practice, for a IL/solvent system, the species represented by i,j cannot span all the atom types, since the full set of partial structure factors is too difficult to be extracted from the measured scattered intensity I(k), and moreover the interpretation of all these data would be equally challenging. Therefore, the information has to be coarse grained, for instance reducing the species to cation, anion and solvent. For neutron scattering, such a coarse division of scattering intensities could be achieved using selective deuteration, i.e., exploiting the large difference in the coherent scattering cross section of H and D. No similar approach is available with X-ray scattering.

In the case of an IL/solvent solution, one is interested, for instance, in the fluctuation of the distribution of cations (+) and anions (−) in space. Therefore, one defines the $S_{++}$, $S_{--}$ and $S_{+-}$ partial structure factors, which can be further combined into a density–density (nn) and charge–charge (QQ) structure factor for the ions, according to:

(4) $$S_{nn}\left(k\right)=\left[S_{++}\left(k\right)+S_{--}\left(k\right)+2S_{+-}\left(k\right)\right]$$

(5) $$S_{QQ}\left(k\right)=\left[S_{++}\left(k\right)+S_{--}\left(k\right)-2S_{+-}\left(k\right)\right]$$

where the former represents correlations in the fluctuations of the total density of ions, while the latter represents correlations in the charge distribution throughout the system. The cross correlations ($S_{nQ}$, see reference [79]) between density and charge often are much less relevant.

Moreover, fluctuations, in the distribution of water in space are quantified by computing the structure factor:

(6) $$S_{ww}=\frac{1}{N_{w}}{\langle \rho_{w}\left(k,t\right)\rho_{w}\left(-k,t\right)\rangle }_{t}$$

where $N_{w}$ is the number of water molecules, and $\rho_{w}\left(k,t\right)$ is computed from the distribution of water molecules in a way similar to Equation (2).

Information on the overall structure of the solution is contained in the low-*k* range (0<k≤0.4 Å${}^{-1}$) of these structure factors, which also provide information on nanostructuring in the mixed phase, whose amplification foreshadows the transition. In this respect, only the $S_{nn}$ of the ions and $S_{ww}$ are relevant, since the low-*k* range of $S_{QQ}$ is strictly constrained by the electroneutrality condition [80]. To first approximation, peaks of $S_{nn}\left(k\right)$ in this low-*k* region point to nano-aggregates of IL in dilute water solutions, as complementary peaks in $S_{ww}$ in concentrate solutions point to water pockets in the liquid salt structure [81].

If the interest is restricted to the mixed/demixed state of the mixture, then the $\lim_{k\to 0}$ of the structure factors carries all the relevant information, and, as a further simplification, the IL/solvent sample can be seen as a pseudo-binary system, made of water and ions, without discriminating between cations and anions. Then, the relevant variables are the fluctuation ΔN of the total number of particles, and the fluctuation Δx in the mutual concentration of ion and water, measured on a portion of the system that is macroscopic without including the whole sample (for which ΔN and Δx vanish) [78].

Referring, for definiteness, to small-angle X-ray scattering (SAXS), the fluctuation 〈Δx〉 can be computed from the k→0 limit of the scattered intensity I(0) according to: [78,79]

(7) $$\frac{I(0)}{N}={\bar{Z}}^{2}\left(\frac{N}{V}\right)k_{B}T\kappa_{T}+{\left[\bar{Z}\delta -\left(Z_{IL}-Z_{W}\right)\right]}^{2}N{\langle \Delta x\rangle }^{2}$$

where *N* is the total number of particles (water and ions, without distinction), *V* the system volume, $\kappa_{T}$ the isothermal compressibility, $Z_{IL}$ and $Z_{w}$ the number of electrons of IL and water, respectively, and $\bar{Z}=x_{IL}Z_{IL}+x_{w}Z_{w}$ their average, weighted by the corresponding mole fractions. Moreover:

(8) $$\delta =\left(\frac{N}{V}\right)\left(\nu_{IL}-\nu_{w}\right)$$

where $\nu_{IL}$ ad $\nu_{w}$ are the partial molar volumes of IL and water, respectively.

Since fluctuations in concentrations and in the number of particles are connected by stoichiometry and by macroscopic relations, once 〈Δx〉 is obtained from Equation (7), the corresponding fluctuation in the number of particles can be estimated as:

(9) $$\left(\frac{{\langle \Delta N\rangle }^{2}}{N}\right)=\left(\frac{N}{V}\right)k_{B}T\kappa_{T}+\delta^{2}N{\langle \Delta x\rangle }^{2}$$

completing the picture on the phase state of the system.

As already stated, besides thermodynamic modelling, the major computational activity on thermoresponsive IL solutions is computer simulation, represented primarily by MD [44] In most cases, MD simulations of molecular fluids are based on empirical force field, describing the potential energy of the system as a function of the atomic positions. Systems consist of molecules, and molecules are defined in terms of atoms and covalent bonds. Many generic force fields are currently used to model systems consisting of water, organic and bio-molecular systems. A few force fields using the same functional form of generic force fields have been re-tuned to represent IL with optimal accuracy [82,83,84,85]. Current force fields often assume rigid, i.e., unpolarizable, ions. The exceptions are a few polarizable force fields [85,86,87], which, up until now, have not represented major tools in IL studies. Compelling evidence for the need of a polarizable force field, especially for mixtures of IL in organic solvents of low dielectric constant, are given in reference [88].

Ab-initio MD, which could provide a more predictive approach and has already been used for IL [89] is still too expensive to deal with the large systems and especially long times required to follow a near-critical phase transition. Moreover, there is no clear indication that equilibrium phase boundaries predicted by ab-initio methods are any better than those given by classical force fields [90] whose accuracy, however, is due to their empirical nature and fitting underlying their parametrization. Quantum chemical computations currently play an auxiliary role in parameterizing, tuning and testing the force field.

On the other hand, coarse-graining, already extensively used for ILs [91,92,93], could greatly help covering the time ad length scales needed for the transition. However, the structural, and directional averaging that is implicit in coarse-graining (see reference [87], page 3) makes it difficult to faithfully reproduce the subtle interplay of geometric packing and of Coulomb, dispersion and hydrogen-bonding interactions that determine the precise position of the coexistence curve in thermal responsive systems.

Coarse graining can be pushed to its limit representing multicomponent solutions in terms of the number or mass density of their different species throughout the system. Classical density functional theory [64], provides the formal framework to deal with this class of models on the continuum, whose relation with thermodynamic modelling has already been pointed out in previous paragraphs.

Mesoscopic models based on dissipative particle dynamics (DPD) or smoothed particle hydrodynamics, representing the system in terms of mesoscopic blobs evolving according to stochastic equations of motion, could provide a useful tool in the chemical engineering of thermoresponsive IL solutions. These models, however, until now have not been used to this aim.

In the thermoresponsive IL/solvent context, simulations require large sizes and long times, therefore are usually carried out using highly optimised and parallel computer packages, running on large clusters and supercomputers. Moreover, most such MD studies are carried out in the NPT ensemble, in which the volume and the osmotic pressures across interfaces are automatically equilibrated. The phase equilibrium problem underlying thermo-responsiveness is particularly suitable for Gibbs-ensemble simulations [94], based on Monte Carlo (MC). Again, up until now, however, not many studies of phase equilibria in IL/solvent systems have been carried out using this method (see, however, reference [95], for an example).

The formation and stability of the interface separating the two components of a biphasic system, whose destabilisation correspond to the onset of miscibility, can be assessed by computing the corresponding interfacial free energy $\gamma_{s}\left(T\right)$ [96]. Assuming that the sample is enclosed into an orthorhombic simulation box, with the (approximately planar) interface perpendicular to the *z* axis, it can be estimated as:

(10) $$\gamma_{s}=L_{z}\left[P_{zz}-\left(\frac{P_{xx}+P_{yy}}{2}\right)\right]$$

where $L_{z}$ is the length of the *z* side of an orthorhombic simulation cell, and $\left\{P_{\alpha \alpha };\alpha =x,y,z\right\}$ are the diagonal (Cartesian) components of the stress tensor, computed from the position of and forces acting on particles during the MD simulation. Then, free energy methods (umbrella sampling, potential of mean force) allow to compute the free energy cost of moving one solute across the interface [96].

What is also in simulation—the structural analysis of homogeneous systems approaching the mixing/demixing line is often carried in terms of partial structure factors. The analysis of radial distribution functions, in principle equivalent, is less transparent than the structure factor route, mainly because in molecular systems many details are blurred beyond the short range of the first few peaks. Hence, the radial distribution functions are useful to zoom on short range aspects such as hydrogen bonding or local coordination of the ions. They may also highlight inhomogeneities on length scales comparable to the simulation box. Features at intermediate scales are less easy to characterise in this way.

A formal framework to analyse MD trajectories is based on Kirkwood–Buff (KB) integrals [97,98], defined in terms of structural data (partial structure factors or radial distribution functions), and providing also non-trivial thermodynamic insight.

In a binary (isotropic) fluid system, the KB integrals $\left\{G_{\alpha ,\beta },\alpha ,\beta =1,2\right\}$ are defined as:

(11) $$G_{\alpha ,\beta }=4\pi \int_{0}^{\infty }\left[g_{\alpha ,\beta }-1\right]r^{2}dr$$

where $g_{\alpha ,\beta }\left(r\right)$ is the radial distribution function for species (α,β), and *r* is the radial distance. One could think of $G_{\alpha ,\beta }$ as a kind of specific absorption between species (α,β). It is easy to recognize $G_{\alpha ,\beta }$ as the limit of the corresponding partial structure factor $S_{\alpha \beta }\left(k\right)$ already defined, since:

(12) $$G_{\alpha \beta }=\frac{1}{\rho_{\alpha }x_{\beta }}\left[\lim_{k\to 0}S_{\alpha \beta }\left(k\right)-x_{\alpha }\delta_{\alpha \beta }\right]$$

Then, given the meaning of $S_{\alpha \beta }\left(k\right)$ in terms of correlations of density fluctuations, it is easy to show that:

(13) $$G_{\alpha ,\beta }=\lim_{V\to \infty }V\left[\frac{\langle N_{\alpha }N_{\beta }\rangle -\langle N_{\alpha }\rangle \langle N_{\beta }\rangle }{\langle N_{\alpha }\rangle \langle N_{\beta }\rangle }-\frac{\delta_{\alpha \beta }}{N_{\alpha }}\right]$$

Partial derivatives of the chemical potential with respect to the number of particles, partial molar volumes and the isothermal compressibility can all be defined in terms of the $G_{\alpha ,\beta }$ [99]. The information on the phase stability of the mixture with respect to demixing is summarised by the parameter:

(14) $$\Gamma_{\alpha \beta }=1-\frac{x_{\alpha }\rho_{\beta }\left(G_{\alpha \alpha }+G_{\beta \beta }-2G_{\alpha \beta }\right)}{1+x_{\alpha }\rho_{\beta }\left(G_{\alpha \alpha }+G_{\beta \beta }-2G_{\alpha \beta }\right)}$$

For a binary mixture, it is relatively easy to show that $\Gamma_{\alpha \beta }$ is directly related to the second derivative of the Gibbs free energy with respect to composition (or, equivalently, to the first derivative of the activity coefficient with respect to composition). Hence, $\Gamma_{\alpha \beta }$ measures the stability of the binary solution. For an ideal solution, in particular, $G_{\alpha \alpha }+G_{\beta \beta }-2G_{\alpha \beta }=0$ and $\Gamma_{\alpha \beta }=1$. Moreover, a solution is stable if $\Gamma_{\alpha \beta }$ is positive, unstable/metastable if $\Gamma_{\alpha \beta }$ is negative. In principle the scheme applies only to homogeneous solutions, and provides a way to monitor the progressive deterioration of the stability with respect to demixing.

The KB formalism is rigorous and it can be used both in experiments, computing the $\Gamma_{\alpha \beta }$ from structure factors, and in simulation, with $\Gamma_{\alpha \beta }$ computed either from the structure factors or the radial distribution functions. Its application, however, requires some care, especially in analysing simulation results. First of all, the KB integrals are defined in the grand canonical ensemble and for an infinite system, at variance from simulations, usually carried out in the NPT ensemble (or NVT, NVE) for finite and sometimes small systems. A variety of algorithms have been devised to correct this drawback (see, for instance, reference [97,98]). Then, the application to IL/solvent systems is confronted with the fact that the system has in fact at least three components, i.e., cation, anion and solvent, and beyond two components the KB formulation is rather involved. More importantly, because of the neutrality condition, the concentration of anions and cations cannot be varied independently, as instead required by the 3-component formalism. Moreover, in this case, however, practical way out of the problem have been devised [100], although, in some cases, of somewhat empirical character. The simplest approach is to consider the IL/solvent system as two-component, treating cations ad anions as indistinguishable. The last comment is that the KB formalism is strictly a reformulation of the same picture contained in the partial structure factors, whose *k*-dependence also provides information on the intermediate length scales, covering nanostructuring, and not only on the macroscopic scale of full phase separation.

Finally, computational research advances knowledge also through idealised models. In the case of LCST, a coarse grained model, able to describe long length and time scales, has been proposed in reference [101], and could be adapted to provide a simple, implicit solvent description of IL/water solutions. The model consists of a binary mixture of isotropic particles interacting through pair potentials. The major portion of the Hamiltonian, written for a system of *N* particles of type A and *N* of type B is:

(15) H^0=∑i∈A,Bpi22mi+12∑i≠j∈AϕAA(|ri−rj|)+12∑i≠j∈BϕBB(|ri−rj|)+∑i∈A,j∈BϕAB(|ri−rj|)

where $\left\{r_{i}\right\}$ are the particle coordinates, and the first term in this equation is the kinetic energy of all particles.

The effect of intra- and inter-molecular interactions on entropy is attributed to auxiliary variables describing *l* harmonic oscillators carried by each particle. Hence, the full system Hamiltonian becomes:

(16) H^=H^0+KEξ+12∑i=1N∑γ=1lmγ,iωi2ξγ,i2

where $KE_{\xi }$ is the kinetic energy of the oscillators, $\xi_{\gamma ,i}$ is the time dependent elongation of the oscillator γ associated to particle *i*, and $m_{\gamma ,i}$ is its mass.

The frequency $\omega_{i}$ of each oscillator is assumed to depend on the local environment in which particle *i* sits, affecting its entropy $s_{i}$ since $s_{i}=k_{B}\left[1+\log\left(k_{B}T/\hbar \omega_{i}\right)\right]$, where $k_{B}$ and *ℏ* are the Boltzmann and Plank’s constants, respectively. To model the LCST of a binary neutral mixture, for instance, one would adopt a binary Lennard–Jones pair potential for particles whose size and dispersion interactions are derived from the atomistic force field. The mixed/demixed state of the system can be measured by the number $n_{i}$ of particles of the same type coordinating particle *i* (homo-coordination), computed up to a suitable cut-off radius: the lower $n_{i}$, the higher is heterocoordination, implying that the system is mixed. To achieve LCST, the system has to gain entropy when the overall homo-coordination of particles decreases. In the model, this is achieved by setting:

(17) $$\omega_{i}=\omega_{0}\left\{\frac{1}{2}-\frac{1}{\pi }\arctan\left[\alpha (n_{i}-n_{0})\right]\right\}$$

**Figure 7:**
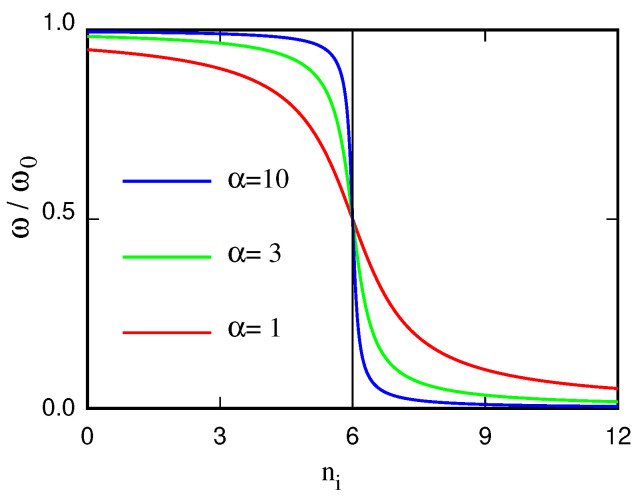
Dependence of the intra-molecular oscillator frequency ω on the local coordination $n_{i}$ (see Equation (17)) causing the LCST transition in the model of reference [101]. The vertical line identifies the reference coordination $n_{0}=6$.

**Figure 8:**
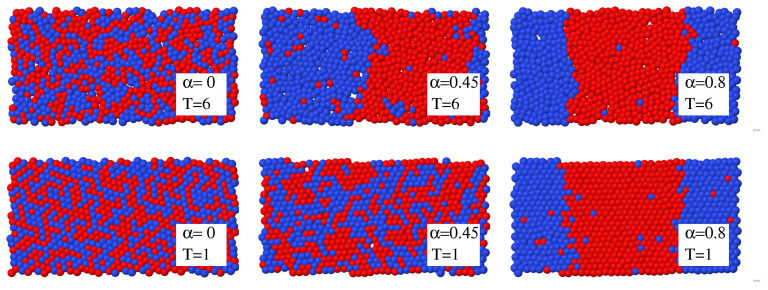
Snapshots of three samples simulated using the coarse grained model of reference [101]. The panels differ in the simulation temperature (T=1 and T=6), and in the value of the parameter α introduced in Equation (17). Reprinted with permission from reference [101].

In this way, the frequency of the oscillators carried by particle *i* decreases with increasing coordination $n_{i}$, resulting in an entropy gain. The constant parameter $n_{0}$ is the coordination number of particles in some reference state, and α determines how quickly entropy changes with changing coordination. Large values of α imply a fast decrease of entropy with decreasing coordination, meaning a high entropy cost due to hydration (see Figure 7). The parameter α, therefore, allows to control the temperature of the transition, decreasing it when α increases. For low values of α, oscillators and particles are nearly decoupled, and the system is mixed at all liquid state temperatures. At high α values, the entropy cost of hydrating particles is high, and the system is phase separated at all liquid state temperatures. At intermediate values of α, the demixing transition occurs in the liquid temperature range, as illustrated in Figure 8 [101].

To model the LCST for IL/solvent systems, one could adopt an implicit-solvent model, representing cations and anions with particle Type A and Type B, respectively. The pair potential would become a charged Lennard–Jones model, and the frequency of the oscillator would again depend on local coordination $n_{i}$, computed up to a radius somewhat longer than the nearest neighbour distance, and counting, in this case, both hetero- and homo-coordination. Using again Equation (17) with the new definition of $n_{i}$ and $n_{0}$, low frequency and high entropy correspond to high coordination at short range, which become favourable at high *T* despite the potential energy cost of expanding the system volume and increasing the ion-ion distance. In the implicit solvent picture, high short-range coordination and low volume imply de-hydration, while the opposite corresponds to hydrated ions of larger effective size.

## 3. Overview of Experimental and Computational Studies

Giving a historically accurate account of the development of a recent and complex research field such as thermoresponsive IL/water solutions is challenging and, in any case, it might be beyond our professional expertise. The historical considerations in this section, therefore, are only tentative, and provided mainly to give a structure to the discussion. The examples discussed in some detail in the present exposition represent a selection among a significantly larger but not vast number of studies on the UCST and LCST of ILs/water and ILs/organic solvents systems. When the historical development was easy to follow, we selected the studies that introduced some novel feature, and impacted later studies of the subject. When it was not possible to follow the historical development, we selected the most recent studies, which give reference to relevant previous studies. reference [18] contains a comprehensive list of thermoresponsive IL solutions that have been experimentally characterised.

### 3.1. Early Studies

Examples of solubility gaps of salts in water and in organic solvents have been discovered long ago, probably by accident, during early electrochemical investigations [102,103] The root of systematic studies of UCST and LCST in IL/solvent and IL/water in particular, lies in the intense activity on critical phenomena of the last thirty years of the 20th century. A paradigmatic study [31] in this area, analysed the miscibility gap of the alkyl-ammonium [N${}_{3444}$][I] ILs in water. A preliminary study [104] considering [N${}_{pppp}$][I] (p≥3) also in water identified a UCST, but the shape of the coexistence curve suggested the presence of a related LCST, whose observation, however, was prevented by crystallisation. This picture was confirmed by measurements on an IL with the less symmetric cation ([N${}_{3444}$]${}^{+}$), decreasing the freezing point and revealing the underlying LCST. As in later papers on thermoresponsive IL/water solutions, the solubility gap, which in this case takes the form of a closed loop (see part (d) in Figure 1), is attributed to the moderate hydrophobicity of the [N${}_{3444}$]${}^{+}$ cation. The critical exponent β is determined for both UCST and LCST, found to be equal in the two cases, and to belong to the 3D Ising class. To achieve the quantitative accuracy required to distinguish 3D Ising from mean-field criticality, the fit of thermodynamic properties has to account for the simultaneous presence of LCST and UCST, separated by 15 °C only.

Similar findings were reported in reference [105] for [N${}_{4444}$][Br] in toluene. Moreover, in this case, the solubility gap is a closed loop in the (concentration *x*, *T*) plane, whose LCST at $T_{c}=297.75\pm 0.05$ K and $x_{c}=0.0270\pm 5\times 10^{-4}$ is below the equilibrium freezing point, but can be identified in the undercooled regime. The results are contrasted with those of the restricted primitive model [23] which only has a UCST. Remarkably, the system lacks hydrogen bonding, which instead is considered a crucial element of thermo-responsiveness, since it provides (through desolvation) the most handy source of entropy required for LCST.

**Figure 9:**
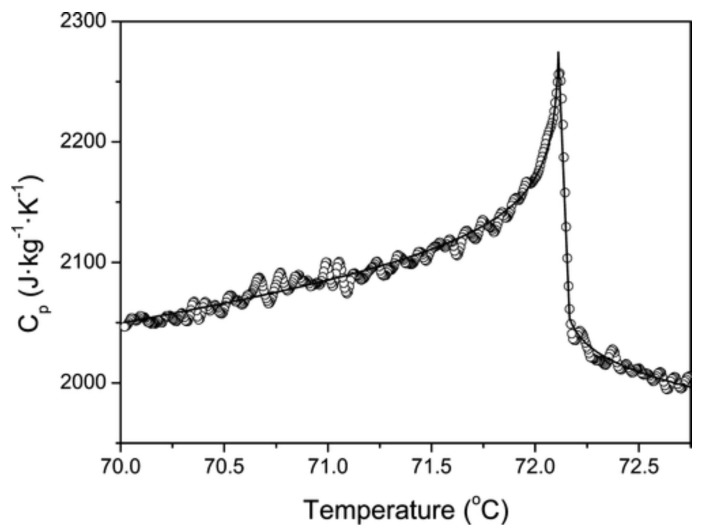
Specific heat anomaly in choline bistriflimide measured by high resolution adiabatic scanning calorimetry. Reprinted with permission from reference [29].

The new wave of interest for thermoresponsive IL/solutions accompanied the grow of the room-temperature ionic-liquid research field, expanding to cover an ever larger number of systems, phenomena and applications [4] In this context, IL/water solutions showing a LCST [106], might have been know for a few years longer than those displaying a UCST [30,107,108]. As already mentioned in the introduction, a very detailed early study of IL/water displaying UCST is reported in reference [29], investigating water solutions of choline bis(trifluoromethylsulfonyl)imide, whose $T_{m}=30$ °C, displaying a UCST at ∼50 wt% composition and $T_{c}=72$ °C. This study is remarkable for the extensive quantitative analysis of properties such as critical exponents and thermodynamic anomalies in the vicinity of the UCST. The specific heat, for instance, has an anomaly (see Figure 9), thermal conductivity κ has no anomaly above $T_{c}$, and below $T_{c}$ the two branches have a difference Δκ which reflects the water and IL relative composition of the two phases.

In the copious new stream of IL-related studies, the first mention of LCST involving a prototypical ILs ([C${}_{m}$mim][NTf${}_{2}$], 1≤m≤5) dissolved in chloroform is reported in reference [109]. Although it does not even mention previous physics literature on similar systems and phenomena [29]. reference [109] in some sense represents a link between the early and present stages of thermoresponsive ILs investigations, since the observation of phase separation is accompanied by a detailed discussion of the phase diagram topology, and of the closed-loop miscibility gap in particular. A related study [110], extends the analysis of [C${}_{m}$mim][NTf${}_{2}$] solubility to other organic solvents (arenes) such as benzene, toluene and α-methylstyrene. In these non-polar solvents, the solubility of the IL increases with increasing length of its alkyl chain. The results of both studies emphasise the crucial balance of solvophobicity/solvophilicity in deciding the location and type of thermoresponsive transition, which sensitively depend on the length *m* of the alkyl chain. This parameter is treated as a continuous variable, exploiting mixtures of cations of different *m*. Mapping the system properties on the phase diagram of idealised models, the authors argue that nano-aggregates consisting of a few IL ion pairs represent the relevant dynamical unit even in the homogeneous phase, and this assumption is supported by the results of electrospray mass spectrometry.

### 3.2. Focus on IL/Water Thermoresponsive Systems

**Figure 10:**
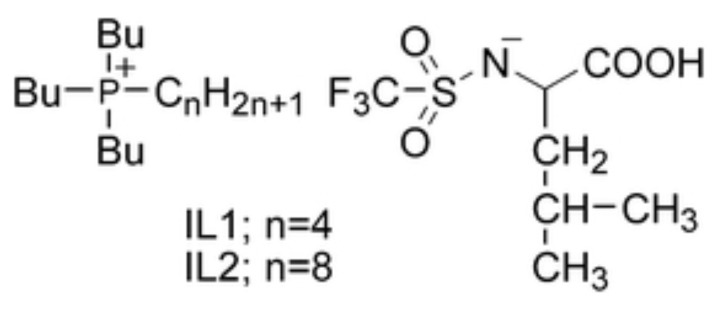
Thermoresponsive ionic liquid consisting of a alkyl phosphonium cation and an amino acid (Leu) anion modified at its amino group to enhance hydrophobicity. Scheme reprinted with permission from reference [113].

In view of applications, IL/water mixtures are of crucial importance, since, besides several other reasons, they avoid volatile and/or toxic organic species such as benzene, toluene, etc. In the context of IL/water mixtures, a series of pioneering papers on LCST have been published by the Ohno group [111]. The first of these studies is a byproduct of their success synthesizing twenty amino acid (AA) based IL (with [emim]${}^{+}$ cation) [112]. To bring the phase separation in the $0\leq T_{c}\leq 100$ °C interval, [emim]${}^{+}$ was replaced with [P${}_{4444}$]${}^{+}$ and the AA anions were made somewhat more hydrophobic by adding a trifluoromethanesulfonyl group to their amino group (see Figure 10) Since several AA-based IL modified in this way display LCST, it was possible to investigate how the phase separation temperature depends reproducibly on the ion structure and water content. In all cases, the observed transition is reversible, and it is not very sharp, since it takes ∼5 °C to manifests itself unambiguously.

**Figure 11:**
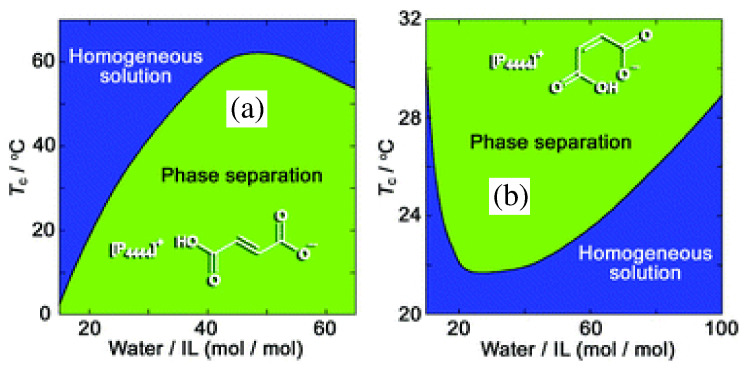
Water content dependence of $T_{c}$ for ionic liquids/water mixtures. (**a**) fumarate; (**b**) maleate. Fumarate and maleate are isomers of each other. Reprinted with permission from reference [114].

A further paper by the same group concerned dicarboxylic protic ionic liquids [114], or, more precisely water solution of [P${}_{4444}$]${}^{+}$ combined with fumarate and maleate anions. Fumarate and maleate are the trans- and cis-isomer of each other, where trans and cis refer to a double C=C bond in their structure (see Figure 11). Despite the identical composition and distribution of single and double bonds, water solutions of [P${}_{4444}$]${}^{+}$ neutralised by either fumarate or maleate have different chemical physics properties, apparently due to an intra-ion hydrogen bond in maleate which makes its charge more delocalised. As a result, for instance, their melting temperature differs by more than 50 °C, and, more importantly for our discussion, their phase properties are different (see Figure 11): fumarate shows UCST, maleate shows LCST. Both for fumarate and maleate, following demixing, the water concentration in the IL-rich phase decreases continuously but rapidly changing *T* away from $T_{c}$ (see Figure 4 of reference [114] for the [P${}_{4444}$][maleate]/water system), as could be read from the solubility lines in the two-phase part of the phase diagram. Seen in reverse, i.e., by approaching $T_{c}$ from the demixed side, the solubility lines describe the increase of water concentation in the IL-rich phase, becoming very rapid (but not diverging, since the overall composition is fixed) at $T_{c}$. Although detailed, this picture says nothing about the mutual structural organization of water and IL, which, instead, might be relevant to understand the transition. Remarkably, ternary mixtures consisting of both fumarate and maleate dissolved in water give solutions which remain mixed over a wide temperature range.

The results of reference [111] suggested that the LCST behaviour of AA-based ILs depended on the dissociation degree of the carboxyl groups on the AA anion. This immediately opened the way to changing the water solubility by changing pH. Adding strong acids or bases to the solution would change the nature of the system, but less disruptive weak acids and bases are already able to trigger the mixing/demixing transition even at constant temperature. In reference [115], such a dual responsive system was achieved by injecting CO${}_{2}$ or N${}_{2}$, whose slight change of pH was sufficient to the task. Moreover, a volatile species such as CO${}_{2}$ can easily be removed from the system, making the transition reversible with respect to both the temperature and the gas-addition stimuli.

A rapidly expanding set of studies on thermoresponsive IL/water solutions soon introduced a variety of other systems, displaying either UCST [5,116] or LCST [117,118]. In general, thermoresponsive IL/water solutions arise from ILs made of weakly polar quaternary phosphonium or ammonium cations with carboxylic (including AA) acid or sulfonic acid anions. The relatively small number of IL systems displaying LCST with water has motivated the successful application of combinatorial chemistry to search for these systems, covering also families of IL not considered in early studies. In reference [118] for instance, ILs were synthesised based on the 1,2,3-triazolium core structure. The sizeable number of systems identified (14%) on a medium–large library (160 compounds) allows to highlight regularities in the composition and structure of compounds having LCST. All systems displaying LCST, for instance, had 11±1 carbon atoms in the alkyl side chains, a regularity possibly reflecting the conditions on hydrophobicity required to show LCST.

A further extension of the field with promising applications concerns water solutions of paramagnetic IL containing the Fe(III) species in the[FeCl${}_{4}$]${}^{-}$ anion [117,119], stable in the high-spin state S=5/2. According to reference [117], water solutions of [bmim][FeCl${}_{4}$] at 50 wt% concentration are demixed at all liquid temperatures. Decreasing the concentration of IL to 20 wt% introduces an entropy-driven demixing, taking place with increasing *T* at $T_{c}=70$ °C. The demixing temperature can be moved up or down in the interval from room temperature up to ∼100 °C by changing IL concentration, or selecting a [C${}_{n}$mim]${}^{+}$ cation of different tail length *n*. Photometry shows that a non-negligible fraction of iron remains in the water-rich phase. These observations are extended and reinforced by those of reference [119], investigating a larger set of 16 paramagnetic ILs containing the [FeCl${}_{4}$]${}^{-}$ anion. The results of dynamic light scattering document the evolution of IL clusters in water, lending support to the picture that nanostructuring and phase separation are related phenomena. The advantage of [FeCL${}_{4}$]${}^{-}$ -based IL is that they contain a metal ion, which is suitable for a variety of catalytic tasks, and are paramagnetic. Following separation, the IL-rich phase can be displaced using in inhomogeneous magnetic field, a procedure that does not apply to the homogeneous paramagnetic-IL/water solution. The disadvantage is that [FeCL${}_{4}$]${}^{-}$ is prone to hydrolysis in the presence of water, although the results of both reference [117,119], suggest that quantitatively this effect is only a very minor one.

Going beyond the simple proof of principle, the Ohno group analysed the structural features controlling the mixing/demixing behaviour, focusing on the role of hydrophobicity [120] Needless to say, the fact that mixing/demixing of IL and water is determined by hydrophobicity is nearly a tautology, whose predictive power depends on the possibility of measuring the hydrophobicity of ILs independently from their miscibility with water. This could be achieved by measuring the partition of the IL in a water–octanol biphasic system [121], but reference [71,120] from the Ohno group define a hydrophobicity index HI by the concentration of water molecules remaining in the IL-rich phase well after demixing. This gives a scale of hydrophobicity/hydrophilicity which agrees with other empirical scales and is also consistent with the predictions of general models, such as COSMO-RS, stating that hydrophilicity of anions is in the order Cl${}^{-}$> Br${}^{-}$> [F${}_{3}$COO]${}^{-}>$ [NO${}_{3}$]${}^{-}>$ [CF${}_{3}$SO${}_{3}$]${}^{-}>$ [BF${}_{4}$]${}^{-}>$ [NTf${}_{2}$]${}^{-}$, while, for cations, ammonium-based ILs are more hydrophilic than phosphonium-based ILs [71].

In principle, the number of water molecules per cation–anion pair in the IL-rich fraction is just an elaboration of the phase diagram, since the proportion of water and IL in both phases can be read from the composition scale of a diagram, such as that of Figure 1. In most systems, in fact, the IL depleted phase is made by nearly pure water. The IL-rich phase instead contains a non-vanishing proportion of water, but in most thermoresponsive IL/water systems, the slope of the coexistence curve is nearly vertical at T≥60 °C; thus, allowing to define HI at the conventional high temperature of 60 °C. As a predictive tool, the model is somewhat self-referring, since to measure HI the compound/water mixture has to be phase separated in the first place. In any case, it is possible to establish a correlation between the HI and the tendency to phase separate, and also to have an estimate of the critical temperature (see reference [71]).

The calibration of the HI index as a predictive parameter has been carried out on IL/water systems based on homologous anions, combined with the same [P${}_{nnnn}$]${}^{+}$ cation, considering primarily water mixtures of nearly equal weight composition. As a validation, the phase diagram of similar ammonium ([N${}_{nnnn}$]${}^{+}$) cations was investigated. The results obtained on many IL/water mixtures show that, as expected, highly hydrophobic ILs, having HI ≤6, form with water stable biphasic systems at all temperatures. On the other hand, hydrophilic ILs form homogeneous solutions again at all temperatures (but HI is undefined). Then, LCST is displayed by water solutions of ILs of moderate hydrophobicity, corresponding to HI∼7. Based on these observations, it was proposed and verified that it is possible to enlarge the set of solutions undergoing LCST transitions by preparing solutes of intermediate hydrophobicity mixing ILs of high and low hydrophobicity. This is in fact an interesting observation, since it provides a further way to tune the properties of thermoresponsive IL towards applications. In fact, in several (optimal) cases, mixing provides a continuous variation of HI, while changing HI by functionalizing the ions provides only discrete jumps. Moreover, by combining different ILs, it is in principle possible to simultaneously tune also other properties, such as the ability to dissolve complex biomolecules, such as cellulose.

Violations of the combination rule just outlined are as interesting as the regularities. It turns out that the solubility in water of mixtures of ILs is not necessarily a linear function of the relative abundance of the ILs (at total IL concentration in water). For instance, according to reference [122], it is possible to prepare an aqueous biphasic system by combining two hydrophilic ILs, which, independently, mix with water at all temperatures. Examples of this behaviour are represented by ILs consisting of the phosphonium cation [P${}_{6668}$]${}^{+}$ combined with amino-acid derived anions, such as [Lys]${}^{-}$ and [Asp]${}^{-}$. The peculiarity is that these AAs have an additional carboxyl ([Asp]${}^{-}$) or amino ([Lys]${}^{-}$) group in their side chain, which causes the formation of strong (anti-electrostatic) anion–anion hydrogen bonds, which drive their separation from water. In these examples, high polarity favours dissolution of macromolecules, hydrogen bonding favours separation from water without decreasing polarity.

Non-trivial effects can be observed as a function of chemical substitutions on a basic molecular body. Benzoate, consisting of a benzene ring and a carboxyl group, which easily dissociates in water, is a hydrophilic anion, and its [P${}_{4444}$]${}^{+}$ salt is fully soluble in water. At first view, adding one hydroxyl or a further carboxyl group on the benzene ring is bound to enhance hydrophilicity. The results of reference [123], instead, show that the effect of functionalization on solubility depends on the ortho, meta- or para- location of the addition. The addition of -OH on the ortho location (salicylate [Sal]${}^{-}$), or of -COOH at the ortho- or meta-position decrease the hydrophilicity of the anion, and cause a LCST transition in the water solution of their salts with [P${}_{4444}$]${}^{+}$. An explanation was proposed in the same paper, in terms of the formation of an intra-molecular hydrogen bond, which curtails the attractive interaction with water.

A detailed further study of two of the systems investigated in reference [120], is reported in reference [76]. The results (obtained by DLS and NMR) highlight the opposite nanostructuring trends in solutions of water/ILs miscible at all temperatures ([P${}_{4444}$][benzene sulfonate])) and those presenting LCST ([P${}_{4444}$][2,4-dimethylbenzene sulfonate]). In fully miscible IL/water combinations, the typical size of aggregates measured by DLS decreases with increasing *T*, thus enhancing the homogeneity of the system. In thermoresponsive systems, instead, the nanostructuring in the mixed low-*T* phase becomes coarser and coarser with increasing *T*, and the size of the water-rich and IL-rich domains becomes macroscopic at $T_{c}$. The local coordination of the IL ions, characterised by NMR on the hydrogen cation, is nearly unchanged across the transition, implying that this is mainly determined by ion clustering even in the water-rich phase. The formation of micelle-like clusters at $T<T_{c}$ and their growth becoming rapid with approaching $T_{c}$ is not the only known picture. In another case [78], nanostructuring is due to the formation of fuzzy (perhaps fractal) structures, whose characteristic lengths increases again approaching $T_{c}$. In both cases (i.e., micellar aggregates and fuzzy clusters) the transition from mixing to demixing is rather gradual, mediated by the change of nanostructuring. Interestingly, the characteristic size of aggregates, which increases rapidly for $T\to T_{c}$ from below, decreases slowly with increasing *T* in the IL-rich phase above $T_{c}$. It would be very interesting to compare these properties with those of systems undergoing UCST. This analysis is not available, either from experiments or simulations.

A closely related system was investigated in reference [124], considering water solutions of a [P${}_{4444}$]${}^{+}$-based IL made with 5-phenyl tetrazolate ([Ph-tet]${}^{-}$]) anion, which should make it more hydrophobic than benzoate, and therefore having a LCST. The results support those from the previous study, showing that water solutions of [P${}_{4444}$][Ph-tet] present a LCST, with a transition temperature ∼10 °C higher than [P${}_{4444}$][Sal] at all concentrations. The number of water molecules in the IL-rich phase well beyond $T_{c}$ is 12 (>7, which is the lower limit), thus confirming the picture of the previous papers from the Ohno group. The replacement of carboxylic group with tetrazolate is one of many pharmacophore replacements that could be used to control hydrophobicity and other properties of ILs.

For LCST, in particular, several experimental studies and simulations (see Section 4 below) emphasised the role of anions, focusing on their ability to form hydrogen bonds, which, in turn, could represent the source of the entropy that drives the LCST transition. The role of cations has been much less investigated, but two studies, in particular, provide useful insight on this aspect [125,126]. reference [125], for instance, applies a 2D hydrophobicity index (instead of the single hydrophobicity parameter HI of reference [71,120]) to quantify independently both the hydrophobicity and the hydrophilicity aspects of solutes. The two indices are defined in terms of suitable derivatives of thermodynamic functions [127]. In addition to providing a more detailed description of hydrophilicity/hydrophobicity, and of their effects on thermo-responsiveness, the method, in particular, allows to estimate the hydration number $n_{H}$ of cations and anions in solution. This parameter counts the number of water molecules strongly bound to the solute ion, not to be confused with the number of solvating water molecules. In other terms, the hydration number is defined in such a way that the ion and its $n_{H}$ hydration waters behave as a unique dynamical entity. The hydration number tends to be higher, and sometimes much higher, for cations than for anions [126]. This number is temperature dependent, and the decrease in hydration water with increasing *T* might represent a source of entropy for demixing as important and perhaps more important than the breaking of anion–water hydrogen bonds. This aspect, not sufficiently analysed until now, will certainly deserve more quantitative investigations in the future.

### 3.3. Non-Water Solvents and Multicomponent Solutions

As already apparent from the discussion so far, UCST and LCST are not exclusive features of IL/water solutions but can be observed in systems made of IL in a variety of organic molecular solvents. Interesting, in this respect, is the study in reference [128], comparing the solubility in thiophene of [bmim][SCN] and [bmim][NTf${}_{2}$], whose temperature dependence displays a LCST for the former, and a UCST for the latter. The analysis of interactions by the experimental determination of the molar volumes and by NMR in the mixed and demixed phases and by MD simulations highlights an attractive interaction between a thiophene proton and the S atom in [bmim][SCN], and a solvophobic character of [bmim][NTf${}_{2}$] in thiophene. These observations point to a difference between the two systems, but do not explain directly why a LCST transition is found in [bmim][SCN] and a UCST in [bmim][NTf${}_{2}$].

In addition to the many studies devoted to a single organic solvent, we point out systematic studies of solubility and thermo-responsiveness of imidazolium-ILs in alcohols [129,130,131], linear and cyclic alkanes [132], and ethers [132]. Taking imidazolium-based ILs dissolved in alcohol as an example, a clear tendency towards UCST behaviour is observed. Moreover, the solubility of IL/alcohol mixtures ([bmim][PF${}_{6}$] in reference [133]) may be greatly affected by the addition of CO${}_{2}$, which, under appropriate pressure, causes the separation of the IL/alcohol mixture into an IL-rich and an alcohol-rich phase, thus providing an effective way to separate the IL from its organic solvent.

The approach of inducing demixing by adding a suitable third species, sometimes represented by relatively simple inorganic salts, has also been used in IL/water solution. The primary aim of the approach has been to create new stable IL and water biphasic systems [134,135], the side result, however, has been to make thermoresponsive even water solutions of hydrophilic ILs that are fully soluble in the binary phase [136]. In all of these cases, the decreased stability of the IL/water solution has been obtained by resorting to inorganic systems, such as K${}_{3}$PO${}_{4}$, having a strong water structuring (kosmotropic) effect, resulting in the salting out of the organic IL. The Hofmeister series provides a first framework to rationalise and predict the effect of simple salts on the thermo-responsiveness of IL/water solutions [137].

A different strategy to control and enhance the thermo-responsiveness of IL/solvent systems consists of adding a suitable organic molecule able to form a host–guest supramolecular complex with one of the IL ions. In the example in reference [74], two macrocycle molecules (pillar[5]arene and a crown ether) added to a thermoresponsive solution of [dmim][I] in acetone affect the LCST in opposite ways; the first one (pillarene) decreases the system $T_{c}$, while the second one (crown ether) increases it. Both macrocycles are known to form a host–guest supramolecular complex with imidazolium ions, and the effect of the crown ether could be rationalised thinking that the sequestration of [dmim]${}^{+}$ reduces the effective IL density, stabilising its mixed state. The opposite effect of pillarene is more difficult to understand, and one can only argue (without independent proof) that the imidazolium incorporation into a -OH rich complex favours its solvation into a polar solvent, such as acetone, despite the contrasting effect of the aromatic side groups.

In some cases, the IL itself could be seen as the additive controlling (and often enhancing) the thermo-responsiveness of non-ionic polymer/water solutions [138]. In reference [138], for instance, adding an ammonium-based protic IL to a water solution of polypropylene glycol of moderate molecular weight endows the system with a great tunability, allowing to bring the LCST demixing temperature at the value most suitable for applications. The practical value of the ternary IL/polymer/water mixture has been demonstrated by the separation of two proteins, i.e., cytochrome c and azocasein segregating them into the IL-rich solution, going from the homogeneous solution at T=25 °C to the phase segregated one at T=45 °C. Remarkably, the protic IL-rich phase contains enough water to retain the protein in its native state, while separation of proteins into pure (non protic) ILs usually involves denaturation. Finally, reference [138] shows that suitable systems can be prepared with only limited (≤10 wt%) IL content, decreasing the cost and limiting possible toxicity effects.

### 3.4. Polymerised ILs

ILs can be polymerised [139], giving poly-electrolytes. Polymerization, of course, requires one or both ions having suitable polymerizable groups, such as vinyl. The resulting polyelectrolytes present remarkable properties. According to the authors in reference [140], these include an *exceptionally high affinity with carbon dioxide, low glass transition temperature, and controllable affinity with water*. LCST in water solutions of poly-ILs has been observed and discussed in this same reference [140]. Moreover, in this case, the important parameter is the hydrophobicity of the IL polymer. As expected, the preparation of poly-ILs with LCST is more likely from monomers that already show LCST [140]. For instance, water solutions of (anionic) polymerised tributylhexylphosphonium 3-sulfopropyl methacrylate, i.e., poly([P${}_{4446}$][C3S]), present LCST demixing at a temperature $T_{c}$, which depends sensitively on water/poly-IL relative composition, but depends only weakly on the polymerisation degree (i.e., molecular weight of the polymer), the difference between the [P${}_{4446}$][C3S], and poly-[P${}_{4446}$][C3S] being 4 °C only. The poly-IL-rich phase at $T>T_{c}$ still contains a non-negligible water fraction. Upon increasing T a few more °C, it undergoes another LCST reversible transition, this time from liquid to gel, which represents a further remarkable type of thermo-responsiveness.

Another early study of thermoresponsive poly-IL solutions is represented by reference [16], reporting that several poly-ILs displaying UCST in water have been synthesised and characterised, consisting of alkyl imidazolium cations dangling from poly(vinyl ether) chains neutralised by [BF${}_{4}$]${}^{-}$. The hydrophobicity of the cation is controlled by the choice of the imidazolium alkyl chains, while the role of the anion has been assessed by replacing water with a variety of organic solvents, resulting in a variety of solubility conditions, ranging from fully miscible or immiscible mixtures at all *T*, to UCST and LCST behaviour in several cases. In the thermoresponsive water solutions, the $T_{c}$ depends very weakly on the molecular weight of the polymer, but increases with increasing polymer concentration, up to the 10 wt% polymer fraction explored in the study. The concentration dependence suggests that the thermoresponsive transition is due more to inter-chain interactions than to intra-chain ones. Moreover, due to the low hydrogen-bonding capability of the imidazolium side groups, Coulomb interactions are likely to be more important than hydrogen bonding for mixing/demixing. The role of Coulomb forces is confirmed by the comparison with the results for other solvents, whose different polarity controls the screening of electrostatic interactions.

A further very detailed study has been devoted to the LCST of water solutions of [P${}_{4444}$][SS] and poly-[P${}_{4444}$][SS] [141], with SS being styrenesulfonate, in which polymerisation concerns the [SS]${}^{-}$ anion. At variance from the previous case, the $T_{c}$ difference between [P${}_{4444}$][SS] and poly-[P${}_{4444}$][SS] (at equal IL/water composition) is sizeable. Moreover, the composition dependence of the demixing temperature is different: it has a minimum at $T_{c}$ in the monomeric case, it decreases monotonically with increasing IL fraction in the polymerised case. These differences point to a different mechanism of demixing. The discussion in reference [141], focuses on the role of IL and poly-IL aggregates in the two cases, which are larger in the polymerised case and require higher temperature to form. The conformation, investigated primarily by different NMR techniques, shows that cations are located at the periphery of the aggregates in the polymer case, and intermixed with anions in the monomeric case. The explanation is somewhat qualitative, but has the merit to rationalise the trends and the differences in the demixing transition temperature.

### 3.5. Reversing the Role of Solute and Solvent

In binary solutions, the distinction of solute and solvent is to some extent conventional, based for instance on the relative amount of the two components or the relative size and mass of the species. Then, the ILs that, up until this point, have been considered the solute in water and in a variety of organic solvents, may be seen as the solvent in different types of thermoresponsive systems, especially those made of non-ionic polymers and ILs. An early example is provided by the thermoresponsive solution of poly(ethylene oxide) derivatives in [emim][NTf${}_{2}$] [142]. Polyethylene oxide (PEO) itself displays a LCST in [emim][BF${}_{4}$] and [bmim][BF${}_{4}$] [143,144], with a critical solution temperature only weakly dependent on the PEO molecular weight. Since the solvent is no longer water but the IL, the range of temperatures of interest is wider, and the $T_{c}$ of these solutions can reach ∼200 °C. The analysis of the experimental studies points to hydrogen bonding between the acidic imidazolium hydrogen and oxygen in PEO, or F in [BF${}_{4}$]${}^{-}$ as the major players in the solubility change as a function of *T*. MD simulations [145], discriminate the role of the two type of hydrogen bonds, arguing that the one with oxygen is more directional and has an entropic cost higher than that with F, thus having a larger role in the demixing with increasing temperature. A recent experimental study extends the analysis to poly(benzyl metacrylate) in 1-butyl-1-methylpyrrolidinium bis(trifluoromethylsulfonate)imide ([BMP][NTf${}_{2}$]) [72], also showing LCST behaviour, with a transition temperature that sensitively depends on the polymer/IL relative concentration, but, again, depends only weakly on the molecular weight of the polymer. The unusual aspect is that, even in the demixed temperature range, the second virial coefficient for the polymer in IL is positive, pointing to good solvent conditions. The θ-point of the mixture, estimated using the Flory–Huggins (FH) model, is significantly higher than the demixing temperature. The deviation between FH, a semi-empirical but often reliable model of phase coexistence of polymer solutions, is a further diagnostic indicator of strong directional interactions between the ions and the polymer, deviating from the smooth and isotropic interactions assumed in the FH approach.

## 4. The Role of Computational Modeling

Given the vast number of potentially thermoresponsive IL/solvent solutions, quick, inexpensive and reasonably reliable predictions of UCST and LCST properties are in great demand. As a result, most of the thermodynamic models briefly outlined in Section 2 have been applied to the prediction of liquid–liquid phase equilibria of ILs dissolved in organic solvents and especially in water. Our review of these investigations is not exhaustive, but aims at giving an idea of what can be achieved by these methods.

An activity coefficient model related to the non-random two-liquid model, neglecting long-range Coulomb interactions, has been used in reference [146], to investigate vapour–liquid equilibria (VPE) as well as LLE in IL/water and IL/organic solvent systems. The model has been parametrised on density data, and on limited solubility data ($\gamma^{\infty }$). For LLE, in particular, the picture provided by the model is qualitatively correct, although not particularly in quantitative agreement with the experimental phase diagrams, especially for the IL/water solutions, with absolute average deviations of ∼10% in the computed composition *x* of the IL-rich and water-rich phases. On the other hand, experimental trends are rather well reproduced, suggesting that the model can be used to highlight correlations among properties of homologous systems (as also shown in reference [147]).

Cubic EOS models have also been used several times to predict properties of ILs and their solutions, introducing a variety of different effects, such as association (CPA) [45], and polarity (PCPA). To the best of our knowledge, only the first (CPA) has been used to compute LLE properties of ILs in water. Moreover, in this case, density and limited solubility data have been used to parameterize the model. The results are somewhat disappointing, with deviations of predictions from experimental data, as declared by the authors, ranging from 4 to 100%.

NRTL and UNIQUAC models have been applied to model ternary solutions of ILs, water, neutral or polar solvents, parametrized on properties of binary systems only [148]. The task is recognised as very challenging, because correlations in the local composition do not satisfy any transitive property, and in fact the results of the models is mediocre, as stated by the authors of the study.

As anticipated in Section 2, models based on the statistical associating fluid theory (SAFT) together with thermodynamic perturbation theory, are more flexible and also more successful than cubic EOS models. In predicting LLE, however, early applications of the model still gave rather uncertain results [58], displaying an unequal degree of success when applied to ILs dissolved in neutral, polar and water solvents. In the water case, in particular, the model in reference [58] gave good predictions for the composition of IL-rich phases, and poor predictions for the water-rich phase. More recent applications of SAFT models [147], however, have achieved much better results, although they are still dependent on the strategy adopted to adjust the model parameters.

In many respects, COSMO-type models represent the most appealing thermodynamic approach to investigate liquid–liquid equilibria and, in particular, UCST and LCST of IL/water solutions, both because of their a-priori predicting capability, and also for their relation with ab-initio methods. Early applications of COSMO-RS (RS ≡ real solvent) [149], predicted LLE properties in qualitative agreement with experiments, although the quantitative results deviate somewhat from experiments, with the discrepancy increasing with increasing hydrophilicity of the IL compound. Representative recent applications are provided in reference [6] (COSMO-SAC, SAC ≡ segment activity coefficient) and in reference [60] (COSMO-RS).

At variance from previous COSMO studies of thermoresponsive IL solutions, dealing primarily with UCST, reference [6] is devoted mainly to LCST systems, covering 256 binary solutions. The IL component is treated in two different ways, considering the ions fully dissociated or fully associated. Once again the results are qualitatively correct, reproducing the thermo-responsiveness of most experimental systems, but quantitatively not so great. Absolute average deviations in the composition of the IL-rich phase are up to 15%, while for the water rich phase, the deviation is about 4%, when assuming fully dissociated ions. The results for the associated ion case are somewhat better, but still not quantitatively correct. The disappointment is mitigated by the fact that no fitting of free parameters is involved in the prediction.

The results of COSMO-RS computations for a statistically significant sample of 181 ILs support the conclusions of all the previous studies, pointing to a qualitative description of the phase equilibrium, and a reliable reproduction of trends along families of compounds.

Atomistic MD simulations of thermoresponsive IL/water solutions have, until now, played different roles from that of thermodynamic models, since MD cannot be used for the extensive screening of hundred of compounds, although MD has contributed significant insight into these systems and phenomena. A clear distinction between the contribution of experiments and simulations to the understanding of thermoresponsive IL solutions is not really possible, at least because several studies combine both approaches (see, for instance, references [32,76,128]). Nevertheless, it is also possible to observe that, in this field, the strength of simulation has been in providing structural details and mechanisms down to the atomistic level. As in experiments, the most MD studies have been devoted to solutions of ILs based on the phosphonium [P${}_{ijkl}$]${}^{+}$ and ammonium [N${}_{ijkl}$]${}^{+}$ cations. Because of the role of hydrophobicity/hydrophilicity (solvophilicity/solvophobicity), most studies include the analysis of hydrogen bonding (see, for instance, reference [76,150,151], and reference [145] for PEO/[bmim][BF${}_{4}$]), determined as a function of *T* and solute/solvent relative composition.

A paradigmatic simulation study might be represented by reference [150], comparing three ionic liquids sharing the common [P${}_{4444}$]${}^{+}$ cation and three different anions, i.e., [CH${}_{3}$OO]${}^{-}$, [CH${}_{3}$OO]${}^{-}$, [PF${}_{6}$]${}^{-}$. The first IL is miscible and the last is immiscible in water at all temperatures. In agreement with experiments, [P${}_{4444}$][CF${}_{3}$COO] has a LCST. The analysis of trajectories points to a rapid loss of water–anion hydrogen bonding with increasing temperature especially for [P${}_{4444}$][CF${}_{3}$COO], suggesting a likely mechanism for the observed thermo-responsiveness. This is supported by the temperature dependence of Coulomb and dispersion energies for cation–anion, cation–water, and anion–water, but, without a quantitative analysis of the system entropy as a function of *T* it is impossible to tell whether the loss of water–anion hydrogen bonding is the cause or the effect of the mixing/demixing transition.

Inspired by the results in reference [122] on controlling the thermoresponsive behaviour by mixing different ILs, the molecular dynamics study in reference [151] investigated water solutions of binary combinations of ILs, having a common cation ([P${}_{6668}$]${}^{+}$) and different anions, selected among [Lys]${}^{-}$, [Asp]${}^{-}$, [Glu]${}^{-}$, [Ser]${}^{-}$, [Ala]${}^{-}$. Mixing different AA anions, which carry -COOH and -COO${}^{-}$ groups in their side chains, increases the number of possible H-bonding combinations (including anion-anion), thus decreasing the affinity of anions and water, and promoting demixing. The increase of this effect with increasing temperature cannot result from the strengthening at high *T* of anion–anion HBs in absolute terms. Instead, the enhancement of anion–anion H-bonding is likely to be a relative effect due to a faster loss of stability of water–anion HBs, because of the entropy penalty of binding water to an anion.

Another prototypical study combining simulation, dynamic light scattering and spectroscopy (NMR, IR, Raman) is represented by reference [76], analysing the microscopic details of the LCST transition in water solutions of tetrabutyl-phosphonium benzene sulfonates. The role of simulation in this study, however, is rather limited, consisting in the computation and discussion of radial distribution functions.

To summarise, simulation has already demonstrated its great value in this field by highlighting structural details and atomistic mechanisms that could not be identified by experiments. However, the application of MD to thermoresponsive IL/water systems and phenomena has until now been affected by severe limitations. First of all, most simulation studies considered LCST IL/water solutions, neglecting UCST systems, while a comparison would be crucial for a deeper understanding. Perhaps more importantly, most simulation studies do not provide much information beyond the local structure around the ions in solution. Moreover, again, in most cases, simulation samples are small, consisting of ∼200 ion pairs and a few thousand water molecules, covering length scales comparable to the size of aggregates in heavily nanostructured systems. The real-space analysis based on the computation of radial distribution functions has the merit of highlighting specific interactions such as HBs. However, it is unsuitable to detect phase separation in its early stages, and unable to provide a direct estimate of the temperature dependent scale of nanostructuring. In a few cases, the analysis of demixing has been based on the temperature dependence of the potential energy, decomposed into its electrostatic and dispersion contributions. This approach is indeed able to detect demixing. However, it is important to remark that, in empirical force field approaches, the decomposition of potential energy into different contributions is to some extent arbitrary, and the analysis cannot be more than qualitative. Last but certainly not least, entropy, which plays a crucial role in LCST, is seldom mentioned and never explicitly computed, although it could be done by thermodynamic integration over a temperature path joining the mixed and demixed phases.

**Figure 12:**
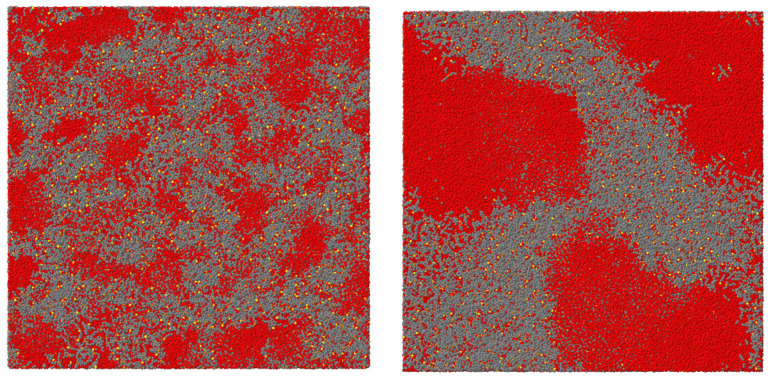
Simulation snapshots showing a 50-50 wt% [P${}_{4444}$][DMBS]/water solution below and above the demixing temperature.

To overcome these limitations, we undertook a large scale simulation study of IL/water mixtures undergoing LCST, covering [P${}_{4444}$][DMBS], [P${}_{4444}$][TsO], [P${}_{4444}$][TFA], [P${}_{4444}$][TMBS], [N${}_{4444}$][TMBS]. For a comparison, we also simulated the water solution of a non-ionic compound (2-[2-(hexyloxy)ethoxy]ethanol), as well as a IL/water solution displaying UCST ([Chol][NTf${}_{2}$]. In all cases, sample sizes have been in the range of $1.2\times 10^{6}$ atoms (L≳22 nm), with simulation times reaching the μs range. Simulation trajectories are still being analysed, but preliminary results show that, in all cases, the combination of MD and empirical force fields is able to reproduce the experimental transition (see Figure 12). The analysis of fluid states in terms of suitable combinations of partial structure factors emphasises the role of nano-structuring. In agreement with experimental data, the simulation results show that the size of aggregates grows with increasing *T*, and the growth is faster ad faster with approaching $T_{c}$ from below. Aggregates, in particular, might be described as micelles, but, because of shape irregularities and some superposition among aggregates, also as a fuzzy distribution of structures covering a few characteristic lengths. Upon demixing, the water phase is nearly pure, while the IL-rich phase still contains a non-negligible amount of water. The analysis of HB shows the crucial role played by them, but the hydration of cations shows similar changes, although, at this stage, it is difficult to conclude whether it represents a cause or an effect. The complete determination of thermodynamic properties is still ongoing, but we plan to compute entropy changes over a wide *T* range.

An interesting contribution, although not framed in the UCST/LCST context, is provided by reference [96], investigating the IL/water interface at room temperature for chlorate and acetate ILs compounds with several alkyl-phosphonium cations of different alkyl-chain lengths. At equal cation, chloride and acetate compounds have similar properties, but the chlorides are slightly more soluble than acetates. Compounds whose alkyl chains are more than a few carbon-long give origin to interfaces with water, but judging from the figures, in most cases, they seem to mark nanostructuring more than genuine bulk phase separation. The temperature dependence of the interface properties, which could provide a different view of phase separation or merging, unfortunately, is not reported. However, the interest of the paper is enhanced by the computation of the potential of mean force to transfer a cation across the interface. This information quantifies the stability of the interface, and, once computed for a guest molecule, it could also characterise the kinetics of transfer across the interface close to a UCST or LCST, which, in turn, might affect extraction and purification processes.

This brief and non-exhaustive review of computational studies of thermoresponsive IL solutions, encompassing both thermodynamic modelling and MD simulations, can be summarised as follows. First of all, the relatively small portion of ILs forming thermoresponsive solutions over the vast number of all IL compounds, drives the quest for extensive screening campaigns, which, up until now, have been the natural playgrounds of thermodynamic models. Among the approaches briefly outlined in the present review, COSMO-like models are the most appealing ones, because their results are at least as accurate as those of competing thermodynamic models, but the parametrization is less open to errors and bias, and does not need extensive and consistent sets of experimental data to be trained.

Molecular dynamics plays a different role, providing microscopic details of structure, bonding, and kinetics that are difficult to obtain by experiments. In this way, MD is greatly contributing to our understanding of thermoresponsive IL solutions. At present, atomistic or near atomistic (i.e., united atom) force field models seem to be the most suitable level to simulate these systems and phenomena. More microscopic models, such as ab-initio MD, are too expensive to cover the system sizes and simulation times of interest, while simpler methods, such as coarse-grained force fields, are not sufficiently detailed to provide quantitative information, although they could represent idealised models for investigating conceptual aspects of thermo-responsiveness [101].

## 5. The Role of IL/Water Nanostructuring

Nanostructuring is a remarkable phenomenon in molecular liquids, particularly in ionic liquids [35,152,153], with nominally homogeneous ILs being sometimes inhomogeneous on the nm scale. This peculiar organisation also appears in IL/water solutions [36,154], in which it refers to the separation of the system into IL-rich and water-rich nanometric domains. Nanostructuring in pure ILs and in IL/water solutions spans a wide range of sizes, and, as discussed below, this is particularly true in the case of thermoresponsive systems. Previous discussions of nanostructuring, based on diffraction and simulation, in most cases cover a size range from 1 to 10 nm. Dynamic light scattering for IL micelles in solution covers a wider range, up to 1000 nm [76] but not many data are available in the IL/water context on sizes above 100 nm.

A decisive contribution towards nanostructuring (instead of phase separation) is given by the amphiphilic character of the IL, which decreases the free energy cost of interfaces. Another contribution might come from the entropy of the inhomogeneous distribution and independent motion of domains, or, more likely, from a favourable curvature-dependent term in the interfacial free energy. A detailed study covering a broad concentration range for a few IL/water solutions is in reference [81]. Nanostructuring has great implications for applications, especially in pharmaceutics and biotechnology [155]. The temperature dependence of nanostructuring can be seen in electron microscopy videos [156]. The systems exhibiting UCST and especially those exhibiting LCST have a marked tendency to nanostructuring in the nominally homogeneous phase and it is tempting to see phase separation as the final stage of nanostructuring with domains growing in size up to macroscopic scale. It is also apparent that the amphiphilic character favouring nanostructuring is closely related to the moderate hydrophobicity invoked for thermo-responsiveness, already suggesting a connection between the two phenomena.

**Figure 13:**
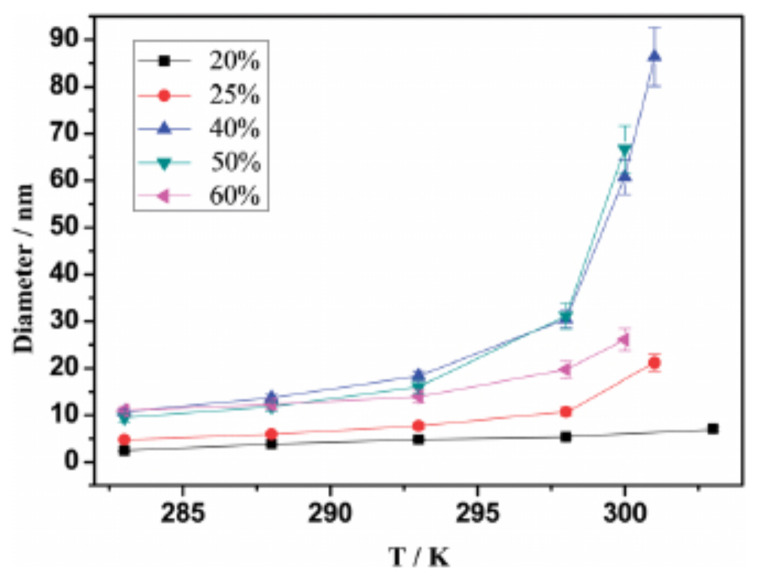
Temperature dependence of the size of [P${}_{4444}$][CF${}_{3}$COO] domains in water for five different IL concentrations in wt%. Reprinted with permission from reference [157].

**Figure 14:**
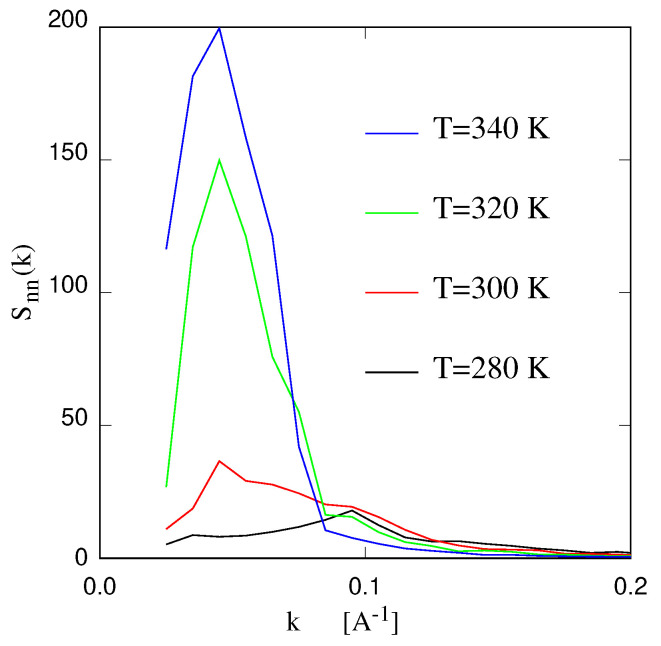
Density–density structure factor $S_{nn}\left(k\right)$ (see Equation (4)) of [P${}_{4444}$][DMBS]/water at 50 wt% composition computed by MD for four temperatures that bracket LCST demixing.

The picture, discussed here for the LCST case, is supported by several experimental and simulation studies. The most explicit statements of the continuity between nanostructuring and demixing are expressed in reference [76,157] (see Figure 13). Further support is provided by our simulation results (see Figure 12), showing a patchwork of IL-rich and water-rich domains in the nominally homogeneous phase at $T<T_{c}$. A more quantitative analysis relies on the low-k limit of the partial structure factors (see Figure 14), which also shows the growth of the characteristic size of domains when approaching (from lower *T*) the transition temperature. Some sort of nanostructuring, whose nature has not been clarified yet, seems to persist in both phases present at $T>T_{c}$, as documented in reference [76] Moreover, again according to reference [76], the characteristic size of the nanostructuring in the IL-rich phase starts to decrease with increasing *T* above $T_{c}$, reversing the trend observed for $T<T_{c}$.

Nanostructured IL/water solutions show an obvious resemblance with nano- and microemulsions [157]. The major distinction is that emulsions are usually ternary systems, made of an oily phase, water and a surfactant. Nanostructured ILs are usually binary systems, in which an amphiphilic IL plays, at the same time, the role of the oily phase and the surfactant. An intermediate case is represented by a pair of ILs dissolved in water, in which the ILs fill two different roles. Using a thermoresponsive IL, it is possible to prepare an equally thermoresponsive micro-emulsion. An excellent example is represented by a mixture of [P${}_{4444}$][CF${}_{3}$COO] and [C${}_{12}$mim][Br] dissolved in water [158]. [P${}_{4444}$][CF${}_{3}$COO]/water is thermoresponsive with a LCST at 30 °C and composition ∼50 wt%, while [C${}_{12}$mim][Br] is a good surfactant. The system has been investigated by experiments, using DLS to characterise the aggregates, and by MD simulation. The analysis of the system properties as a function of composition highlights the typical swelling properties of microemulsions. The analysis of temperature effects allows to investigate the mechanism of de-emulsification, which takes place at low temperature (∼15 °C), due to the interplay of hydrogen bonding between cation–anion and anion–water. Perhaps more importantly, the paper shows that the addition of a minority IL component to the binary system, playing the role of the surfactant, i.e., decreasing the free energy cost of the interface, could greatly extend the range of thermo-responsiveness, and improve the control of this crucial phenomenon. Of course, thermoresponsive microemulsions could find useful applications in the same areas now under considerations for ILs, including catalysis, oil recovery, pharmaceutics, and drug delivery, in particular.

## 6. Applications

The focus on applications, with their extended cycling between temperatures and chemical conditions and their economic and environmental constraints, requires the careful considerations of chemical stability and reactivity [159]. We disregard issues of thermal and electrochemical stability because up to 100 °C thermal decomposition is not quantitatively important for the species considered in this paper, while electrochemical stability is not a concern for the applications covered by the review.

Ionic liquids are usually described as chemically stable, but, of course, this statement has exceptions and limitations. This is particularly true in the case of IL solutions in water, which can drive hydrolization processes [4,160]. Another important degradation process, especially important for cations, is photo-oxidation, which, however, besides negative effects, could also have a positive role in removing residual ILs in waste products.

The most obvious problematic case is represented by fluorinated ILs, based on anions such as [BF${}_{4}$]${}^{-}$ and [PF${}_{6}$]${}^{-}$, which are prone to hydrolysis and produce HF, which is toxic and corrosive [4]. The hydrolysis rate depends also on the cation, with long-alkyl chain imidazolium ions being far from optimal in this context [161]. According to reference [162], however, other fluorinated species such as [NTf${}_{2}$]${}^{-}$, [(C${}_{2}$F${}_{5}$)${}_{3}$PF${}_{3}$]${}^{-}$, or [H(C${}_{2}$F${}_{4}$)SO${}_{3}$]${}^{-}$ are practically stable with respect to hydrolysis, having a half-life of 1 year at ambient conditions, and showing significant degradation only after several days at T=50 °C in water solutions of pH ranging from 1 to 13. On the other hand, other promising thermoresponsive IL compounds, based on methyl-, ethyl-, butyl-, and octyl sulfates undergo hydrolysis at ambient conditions, although over times of several days [163]. As already mentioned, hydrolysis problems also affect the thermoresponsive solutions of magnetic ionic liquids based on [FeCl${}_{4}$]${}^{-}$.

To summarise, the chemical stability of IL compounds in thermoresponsive solutions certainly raises a series of concerns. These are amplified when considering real-life applications, because the unavoidable presence of impurities greatly expands the range of possible reactions, whose products could also affect the environment or the general safety of the process. However, there seem to be still a range of compounds and conditions that could support a variety of applications besides those in separation, extraction and purification, or catalysis that are already relevant in industrial and technological settings [38,39,40].

### 6.1. Extraction and Separation, Catalysis

Extraction and separation are widely used chemical processing techniques often based on the usage of liquid biphasic systems, and aqueous biphasic systems (ABSs), in particular. In this domain of applications, ILs have already gained an important foothold, whose state of the art is summarised in reference [7,164]. In this context, UCST or LCST add more control and selectivity, and can greatly improve the kinetics of the process. Liquid–liquid extraction, in particular, can take place both through UCST [165,166] or LCST [113]. The process applies both to the recovery of IL from water or to the extraction, purification and separation of biomolecules from solutions. An exemplary early application in this domain is the extraction of the cytochrome c (Cyt. c) protein from a water solution [113] exploiting the LCST of [P${}_{4444}$][Tf-Leu]/water mixtures, where [Tf-Leu]${}^{-}$ is the trifluoromethanesulfonyl leucine anion, derived from the leucine amino acid. According to reference [113], the IL/water mixture is homogeneous at T=20 °C and inhomogeneous at T=25 °C, and the extraction process, or, in other terms, the transfer of Cyt. c from the water phase to the IL phase is simple. First, the water solution with Cyt. c is mixed with the IL at $T<T_{c}$, giving a homogeneous liquid mixture. Then, *T* is raised above T=25 °C, resulting in a phase-separated system, in which Cyt. c is found predominantly in the IL-rich phase, which also contains ∼20 wt% of water. The residual water content in the IL-rich phase is instrumental in enhancing the protein solubility, and preserving the protein native state. The crucial parameter in the extraction process is the partition ratio of Cyt. c between water and IL in the phase separated state at higher *T*. For any given protein (such as Cyt. c) the ratio depends on the IL choice. In the [P${}_{4444}$][Tf-Leu] case, less than 0.1% (representing the lower detection limit of the UV-Vis spectrometry measurement) Cyt. c remained on the water side of the biphasic system. This value is an equilibrium value, determined by the different solvation free energy of Cyt. c in the two solvents; hence, it does not depend on the presence of the LCST at lower temperatures. However, mixing the initial water solution of Cyt. c with IL below the LCST point greatly enhances the kinetics of the transfer from water to IL, with positive effects on the viability of the extraction approach. As expected, the partition varies according to the protein, as shown in reference [113] by measuring the partition of several haem and non-haem proteins, displaying a full range of behaviours, from virtually complete transfer from water to [P${}_{4444}$][Tf-Leu] to protein segregation into the water-rich phase, depending on the electrostatic charge and hydrophobicity.

A similar approach, based on biphasic IL/water systems with a reversible LCST, has been followed in later studies to extract a number of other proteins [167], but also simpler organic and biomolecules, fragrances, and cosmetics [168], as well as drug-like molecules [169]. Thermoresponsive IL are also promising in view of extracting oily species from microalgae for biodiesel production [170], or to remove noxious sulfur species from hydrocarbon fuels [171].

Another major task for thermoresponsive IL/water mixtures is the extraction of heavy ions from water [166,172]. The aim is both to refine valuable rare earths [173], as well as to extract a variety of metals from drinkable water. As an example, a water solution of [Chol][NTf${}_{2}$] with a UCST of 72 °C has been used in reference [174] to extract Nd(III). The extraction process is enhanced by adding a suitable extractant, represented by a chelating compound able to sequester Nd(III) into a tight complex more soluble in the IL than in water. In a final stage, Nd(III) is recovered from the IL-rich phase with a concentrated HNO${}_{3}$ solution. A special niche application of thermoresponsive IL solutions is the recovery of valuable metals from electronic waste [40].

Separation by thermoresponsive IL-solvent systems has a special application in catalysis. Moreover, in this case, UCST and LCST are used to improve the kinetics of the catalytic process, carrying out the relevant chemical reaction in the homogeneous phase, while extracting the products and recovering the (often expensive) catalyst upon crossing the demixing line towards the two phases regime. The practical importance of this application is large and growing rapidly. As a consequence, the corresponding literature is overwhelming. Therefore, we refer to a recent review [18], which is more chemistry-oriented than our chemical physics view of the field.

### 6.2. Water Desalination and Purification by Forward Osmosis, and Water Harvesting from the Atmosphere

The demand of non-conventional fresh water to meet the ever growing needs of humanity is so apparent that it does not need to be discussed in this review. Driven by these needs, desalination of seawater (salt content from 3 to 4%) and brackish water (from 0.5 to 3%) has become a relevant player in the global water market, with about 100 million m${}^{3}$/day desalinated water produced per day. This amount, however, still represents about 1% of the total fresh water consumption, hence desalination still has an enormous margin of growth. Current desalination methods rely on: (1) distillation, in which water is vaporised by heating (possibly combined with a sudden pressure drop in flash distillation) and then re-condensed, and (2) reverse osmosis (RO), in which water is pushed through a thin membrane by the application of mechanical pressure, which is needed to overcome the osmotic pressure. Both methods, however, are energy intensive, with the most efficient one (RO) currently requiring around 2 kWh per cubic meter of fresh water [175]. Additional problems faced by RO are due to the sizeable applied pressure, which causes the wear and fouling of the membrane.

**Figure 15:**
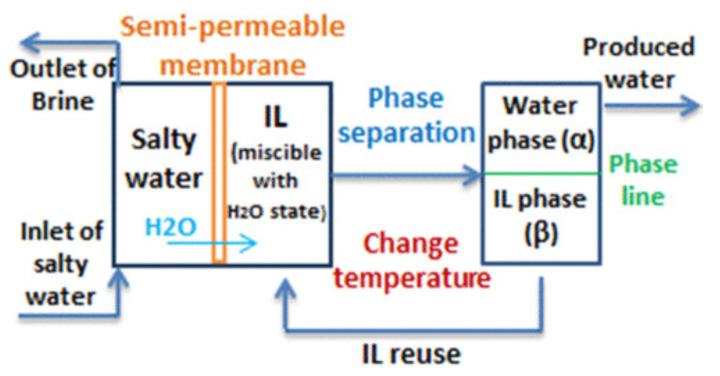
Scheme of principle for FO desalination. The change of temperature required to recover the draw solution is positive with LCST solutions, negative for UCST solutions. Reprinted with permission from reference [42].

An appealing alternative might be represented by forward osmosis (FO) [176,177], which, similar to RO, relies on the filtering by a thin membrane. A few key choices, however, may give an edge to FO with respect to RO, although achieving this advantage still requires significant development. In the first FO step (see Figure 15), water flows spontaneously through the semipermeable membrane from the saline feed solution to a so called draw solution, which, at the beginning, consists of a dry hygroscopic compound. The flow continues until the osmotic pressure is equalised on the two sides of the membrane. Hence, this step virtually does not require any energy input, since the osmotic pressure replaces mechanical pressure in driving the flow of water through the membrane. Since at this stage filtration does not require the application of external pressure, this step is less prone to wear and fouling of the membrane than in RO. Moreover, since IL ions are of medium–large size, back diffusion of IL ions into the feed solution is limited. The second step, however, consist of the separation of water from the draw solution, at the same time regenerating the draw solute, which can be reused to continue the process. In principle, this second step is again a desalination step (IL being the salt) from a solution whose osmotic pressure is even higher than the feed solution. Hence, FO cannot be as energy efficient as the best competing desalination process. In practice, the requirements of high osmotic pressure for the first step and easy separation for the second at first seem contradictory. Resorting to a thermoresponsive solution, and to a thermoresponsive IL/water solution in particular, allows carrying out the separation at the cost of a moderate (10–20 °C) increase of temperature, taking place not far from room temperature (while most distillation plants operate at T>150 °C). In this way, the separation of product water and the regeneration of the draw solution can be largely carried out using low-grade heat that can be represented by waste heat from industrial processes or provided by solar power with no or limited concentration. Hence, on the long run, FO might represent the most cost-effective alternative. Moreover, FO is suitable also for small installations, serving isolated communities in less economically favoured regions. The separation of the draw solution into water and solute, which is then recovered and reused, can take place either upon cooling or heating. The second possibility is the preferred one, since in this case the feed solution and especially the delicate polymeric membrane separation operates at room temperature and only the draw solution needs to be moderately heated. Following this FO route, therefore, demixing takes place through an entropy driven transition, with the draw solution crossing on heating its so-called lower critical solution temperature (LCST).

Before reviewing FO desalination applications, we should remind the reader that the molarity of sea water is 0.6 M of NaCl, corresponding to an osmotic pressure of about 27 bar. The minimum theoretical energy required for desalination computed from thermodynamic functions is 1.09 kW/m${}^{3}$. Important parameters for a FO installation are the osmotic pressure of the draw solution, the flux of water that can be achieved through the membrane, and the reverse diffusion of draw solute into the feed water, which could impose frequent addition of (expensive) draw solute to the system. As a proxy for the osmotic pressure, one can use osmolality *m*, i.e., the number of particles and ions in particular (converted in moles through the Avogadro constant) per Kg of solvent. An approximate value of the osmotic pressure is obtained as π=mρRT, where ρ is the density of the solution, and *R* is the molar gas constant. Osmolality of electrolytes is increased by dissociation, and decreased by ion association. Therefore, one is confronted again by a nearly contradicting requirement, since to increase molality one should achieve good dissociation, but good dissociation, corresponding to hydrophilic conditions, might prevent demixing and thermo-responsiveness.

As already stated, FO desalination could be based both on UCST and on LCST demixing, with a preference for the latter. Remarkably, the two major papers introducing thermoresponsive IL to FO desalination made a different choice on this aspect, with reference [43] relying on LCST, and reference [42] relying on UCST. reference [43], in particular, compares the performance as draw solutes of three IL, i.e., [P${}_{4444}$][2,4-dimethilbenzenesulfonate] ([P${}_{4444}$][DMBS]), [P${}_{4444}$][mesitylene sulfonate] ([P${}_{4444}$][TMBS]) and [P${}_{4444}$][Br]. Of these compounds, [P${}_{4444}$][DMBS] at 70 wt% has the best drawing performance, while [P${}_{4444}$][Br] has no practical ability, i.e., has insufficient osmotic pressure, to draw water from saltwater. Both [P${}_{4444}$][DMBS] and [P${}_{4444}$][TMBS] have a potential for applications. Their LCST demixing points vary from 30 to 50 °C, depending on concentration. Even for [P${}_{4444}$][DMBS] the drawing ability is a sensitive function of temperature. At 14 °C (that might be too low for several applications), a 70 wt% solution draws water from a 1.6 M NaCl solution, which is 2.7 times more concentrated than seawater. Considering seawater as the feed solution, expected operational parameters for real-life application could be: pristine draw solution of [P${}_{4444}$][DMBS] at 70 wt%; diluted solution after water sorption at 30–50 wt%. Heating ∼16 °C above the LCST demixing point regenerates the draw solution at a level that can be reused without further processing, while the water rich phase contains less than 10 wt% IL, with an osmotic pressure of about 6 bar. This last fraction has to be purified by nano-filtration or RO (but operated at low pressure and requiring less energy), recovering the residual IL and producing high-quality fresh water.

A detailed estimate of the energy required for the process shows that the electrical energy consumption (for the final RO step, for instance), is much below (about 16%) the minimum thermodynamic value of 1.09 kWh/m${}^{3}$. The full desalination process requires a larger amount of thermal energy (for the first demixing step), bringing the total above the thermodynamic minimum, and above current RO values. Because of the low demixing temperature of thermoresponsive IL/water solutions, however, the heat can be provided by waste heat or by solar irradiation, as already stated. The flow of water through the device, which is a further important parameter, is comparable to that of high-performance RO desalination. Higher capital investment, however, can be required.

Promising results were presented also in reference [42], investigating the FO performance of a draw solution based on a protonated betaine cation with [NTf${}_{2}$]${}^{-}$ anion ([Hbet][NTf${}_{2}$]), which has a UCST of ∼56 °C. In this case, the pristine draw solution has 88 wt% salt concentration, and the sorption step takes place at 60 °C. Regeneration of the draw solution takes place on cooling to room temperature, with an overall performance comparable (and in some aspects even better) to that of reference [43]. For instance, the osmotic pressure of the draw solution is such to draw water from a 3.0 M NaCl solution. Moreover, in this case, the regenerated draw solution could be used without further processing, and the water-rich phase requires further purification. The energy balance is less detailed than the one given in reference [43], and the thermal energy requirement (44.15 kWh/m${}^{3}$) seems to be relatively large. However, the study is meant to provide a first proof of principle of FO using UCST draw solution, and several choices might not have been carefully optimised.

A recent paper that might represent the state of the art to date [178] focuses on the same class of phosphonium ionic liquids of reference [43], i.e., [P${}_{4444}$][DMBS] ($T_{c}=36$ °C) and [P${}_{4444}$][trifluoroacetate] ([P${}_{4444}$][TFA], $T_{c}=31$ °C), but implements a different heating approach. Perhaps, more importantly, it introduces a lab-scale prototype and a testing protocol. Heating, in this case, is achieved by a photonic heater, which converts solar radiation into black body radiation whose intensity peaks in the mid-infrared, efficiently absorbed by the solution. This non-contact heating increases the thermal efficiency, thus improving the overall energy balance, bringing FO with ILs one step closer to break even with RO. In the testing stage, pristine draw solutions at 70 wt% IL concentration are diluted to 40–50 wt%. The diluted solution is heated ∼20 °C above the LCST, obtaining again a draw solution at 70–80 wt% concentration, and water in which the IL concentration is <10 wt%, to be processed by nanofiltration or low-pressure RO.

In between the early and latest papers, a few studies analysed a variety of aspects in the FO process. reference [179], in particular, analyses a few more ILs than in the other papers, and discusses the relation of osmolality and hydrophobicity of the ILs. In the limit of vanishing concentration, the osmolality of an [M][X] electrolyte is two times its molarity. With increasing concentration, even weakly hydrophobic ILs will form aggregation, decreasing osmolality below its ideal (fully dissociated) value. This, in turn, decreases the osmotic pressure, which is the first most important parameter of the draw solution. Hydrophobicity, however, affects a variety of other properties, such as the occurrence of demixing, the surface tension and viscosity. reference [179] confirms the clear correlation of all these properties. Moreover, it emphasises the role of the critical micellar concentration, i.e., the concentration at which IL aggregates start to form, which marks the appearance of anomalies in all the properties listed in the previous sentence. The interest of reference [179] is that it shows directions to explore to optimise ILs as components of draw solutions, although, at the same time, it shows that the common relation with hydrophobicity poses constraints on the property optimisation that can be achieved.

Several very recent papers might mark a renewed interest in the subject [180,181,182]. An urgent problem that needs to be addressed in future studies is the relatively low water flow achieved until now by thermoresponsive IL/water desalination set ups.

Arsenic contamination of ground water is a major health hazard in several countries (for instance, Bangladesh, China, India, Nepal, Argentina, Mexico) and methods to decrease its concentration in drinking water are in great need. Brackish water suitable for FO desalination may also be affected by high arsenic content, and unfortunately present FO membranes are not effective in preventing its transfer to the product water. An approach being developed relies in functionalizing FO membranes by the imidazolium group, able to increase the membrane selectivity in containing cationic arsenic species [183]. Another approach, not discussed in the literature, but that could be envisageable, consists in performing desalination and arsenic sequestration in the same FO demixing step, confining As in the IL-rich phase. Such an approach would require also a way to extract arsenic from the IL before re-using it as the draw solution.

**Figure 16:**
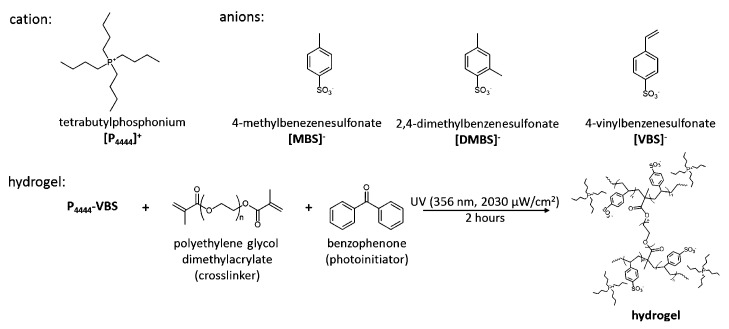
The [P${}_{4444}$]${}^{+}$ cation and the three arene anions considered in the preparation of the IL-hydrogel hybrid thermoresponsive draw solution for FO desalination [184]. The hydrogel was obtained by polymerising [P${}_{4444}$][VBS], cross-linking the polymeric chains through a suitable linker, as shown in the figure. Reprinted with permission from reference [184].

An open direction for improving the FO performance of thermoresponsive IL/water solution is based on the usage of a hybrid IL-hydrogel mixture as the draw solution [184]. The chemical composition and schematic structure are indicated in Figure 16. The IL ([P${}_{4444}$]${}^{+}$ coupled to one of the three anions shown in Figure 16) is similar to those discussed in the previous paragraphs of this subsection. The hydrogel was obtained by polymerising [P${}_{4444}$][VBS] through the vinyl group on the anion, and producing a 3D mesh in water by a suitable cross-linker (see Figure 16). The resulting system retains the thermo-responsiveness of its constituents. The advantages, resulting from purely materials science aspects, include: a more durable draw solution; enhanced purity of the produced water; the possibility of devising a continuous FO process. Since this subtopic is somewhat removed from the main subject of our review, we refer to reference [184] for all the details.

A qualitatively new technology named *directional solvent extraction* (DSE) does not require membrane filtration and is based on oily solvents (including ILs)[185], which do not dissolve in water, but absorb water and repel simple salts, such as NaCl. To separate water and its *directional* solvent, this approach also relies on strong variations of the relevant solubilities, which, however, do not need to "rich" the stage of a genuine UCST or LCST transition.

We should note that FO devices could be used for drying biomolecules and in food processing. Moreover, in this case, the advantage of a thermoresponsive IL would be that of easily regenerating the (possibly expensive) draw solution, although in this niche, application of energy considerations might be less pressing than in large scale desalination.

While seawater desalination is already a reality, and it is likely to greatly expand in the coming decades, it is also apparent that it cannot meet the needs of vast arid regions far from the sea and from other sources of salty and brackish water. Even in the desert, however, the relative saturation of water in the atmosphere can reach 40% during the night, and could be captured by suitable sorbent materials to be released during the day [186]. Water harvesting from the atmosphere following the approach just outlined has been proposed and implemented using a variety of sorbent materials, including zeolites, metal-organic frameworks, hygroscopic salts, desiccants, such as silica gels [187]. Both ionic liquids [188] and thermo-responsiveness (of polymers and gels) [189,190], have been separately considered for this task, but to the best of out knowledge thermoresponsive ionic liquids have non been tested as sorption materials for atmospheric water harvesting. It might be worth it to analyse this application in more detail in the future, since the rapid variation of solubility with temperatures at relatively low water concentrations of thermoresponsive IL/water systems could help, especially at conditions that prevent the usage of different methods, such as moisture and dew collection.

### 6.3. Heat Storage in Thermoresponsive IL/Water Systems

The intermittent operation of renewable power generation sources, such as wind and solar energy, has greatly increased the need for large scale energy storage. A popular approach to store thermal energy relies on the high specific heat capacity of molten salts, and especially on the latent heat of phase–change materials. In this context, ILs have been considered several times as promising systems, because of a favorable combination of properties, including a relatively low operational temperature (suitable for domestic applications), moderate environmental risk and low flammability [191,192,193,194].

We should note that the mixing/demixing transition of UCST and LCST IL/water mixtures represents an additional example of phase change that could be exploited for heat storage and, the LCST case in particular, has a specific advantage for long term storage. Let us consider this latter case. Starting from room temperature, at which the IL/water solution is stable and homogeneous because of lower enthalpy, increasing *T* progressively increases the role of entropy, stabilizing the de-mixed state, whose enthalpy is higher, but whose entropy is higher too. Physical separation of the two phases (inserting a diaphragm, for example), possibly favoured by the different density of the water-rich and IL-reach fractions, permanently traps the system into a demixed state, preventing its degradation through mixing when T decreases below $T_{c}$. No mixing enthalpy can be lost after separation, but the mixing enthalpy can be recovered as heat by mixing again IL and water. In principle, any temperature reversible chemical reaction could be used to the same aim, but the IL/water de-mixing has crucial advantages, because mixing/de-mixing is completely reversible, the IL and water can be recycled indefinitely, many IL are not particularly dangerous for the environment, and $T_{c}$ is only moderately beyond room temperature, allowing again the exploitation of low grade heat.

**Figure 17:**
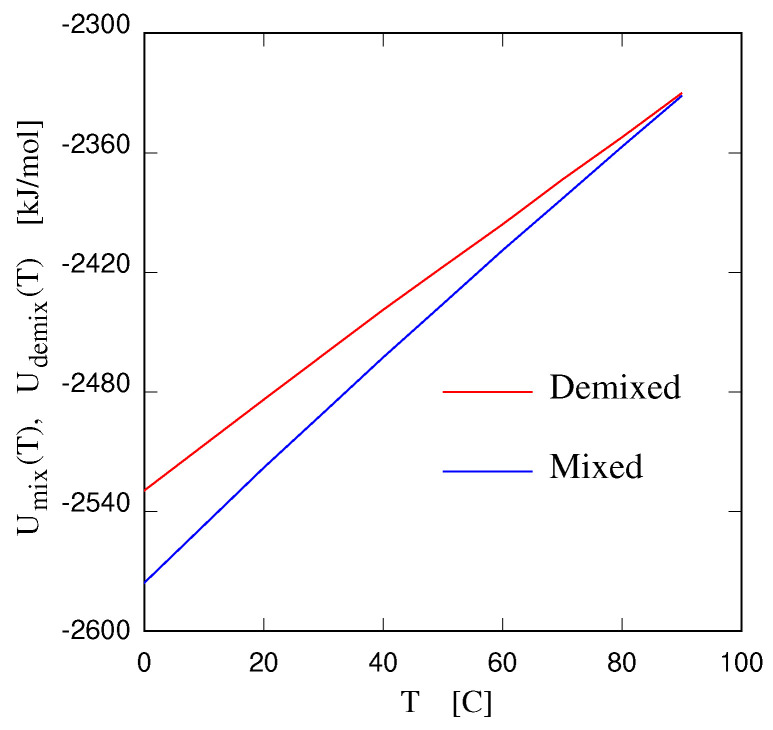
Potential energy of [P${}_{4444}$][DMBS]/water systems at 50 wt% composition from MD simulations. Blue line: unconstrained system, mixed at low *T* and demixing with increasing *T*. Red line: system whose biphasic state is retained by physical separation at all *T*.

To validate this hypothetical picture, we re-analysed the data of our MD simulations, comparing the temperature dependence of the potential energy $U_{mix}\left(T\right)$ of the [P${}_{4444}$][DMBS]/water solution undergoing LCST phase separation with the $U_{demix}\left(T\right)$ of equal amounts of water and IL simulated independently over the 0<T<100 °C interval. The results are reported in Figure 17. The two curves (nearly but not quite straight lines) cross at height *T*, since in this case even the combined IL+water sample consist of two separated phases. At the lowest temperature T=0 °C, however, the two curves differ, and their difference $\Delta U=U_{demix}\left(0\right)-U_{mix}\left(0\right)$ is the mixing energy of [P${}_{4444}$][DMBS] and water at 50 wt% composition and T=0 °C. This difference ΔU represents the amount of heat that can be permanently stored in the demixed system. As already stated, it can be recovered simply mixing again IL and water. Admittedly, in the system, we simulated ΔU(T=0) is relatively small (only 10%) compared to the potential energy difference $U_{mix}(T=100\;{}^{\circ}$C$)-U_{mix}(T=0\;{}^{\circ}$C) to be provided in order to drive the system from the low-T mixed state to the high-T demixed state. However, the appeal of the approach we propose is increased by considering that also the heat that is not permanently stored does not need to be wasted, but can be recovered on a shorter time scale for a variety of heating purposes. Moreover, the estimated ΔU(T=0) quoted above is only the the result for the first IL we tried, and certainly there is a margin for improvement. The enthalpy associated to nanostructuring has already been factored into the heat storage capability of ILs, but the progression from nanostructuring to phase separated has the advantage to make permanent part of this storage capacity [195,196].

## 7. Summary and Discussion

Thermoresponsive IL/water solutions consist of systems with a mixing/demixing transition located in the liquid–water range ([0−100] °C) of temperature, taking place with decreasing *T* in systems with a UCST (see Figure 1b), or with increasing *T* in systems having a LCST (see Figure 1a). A few systems display both a UCST and a LCST, having a closed-loop solubility gap in their phase diagram on the (composition, *T*) plane. In the IL/water case, systems with UCST seem to be more numerous than those with LCST. This observation, however, is only empirical, it might reflect our limited knowledge of the phase diagram for the vast multitude of IL/water systems, and, in any case, the prevalence is reversed in other related systems, such as IL/polymer or poly-IL/solvent systems. Again, for the IL/water case, the UCST and LCST points tend to occur at nearly equal composition (50-50 wt%) in IL and water.

In the known cases, thermo-responsiveness occurs in systems such that the hydrophobic and hydrophilic character of the IL solute, usually associated to different chemical groups, nearly compensate each other, making the IL marginally soluble, with a mixing/demixing that is sensitive to *T* over the limited 0<T<100 °C range. The chemical physics properties of these systems have been probed mainly by differential scanning calorimetry, able to identify phase changes in the solution; by light, neutron and X-ray scattering, highlighting the *T*-dependent nanostructuring in the nominally homogeneous phase; by 1D and 2D NMR, probing the local environment of different species, their spatial arrangement and the relaxation channels among them; by molecular dynamics simulation, providing a microscopic view of structure and bonding, as well as kinetics and mechanisms of the thermoresponsive transition. All these techniques together emphasise the role of the anions, and, in most cases, of their hydrogen bonding with water, cations and among themselves. In the LCST case, in particular, entropy is the eventual winning side of the competition with enthalpy, driving demixing with increasing *T*. The decrease in the number of hydration water molecules, tightly bound (primarily) to anions by hydrogen bonds is the most apparent mechanism for the entropy increase and related demixing at high *T*.

A recurring theme in this field is the relation of nanostructuring in the one-phase stability range and phase separation, which is emphasised by a number of experimental studies and especially by simulation. Moreover, it is apparent that the demixing transition is rather continuous, even away from the single critical point at UCST and LCST. However, it is not clear yet whether nanostructuring over increasingly wide domains is a general and necessary feature of thermo-responsiveness.

The broad experimental exploration of thermo-responsiveness of ILs has shown examples of IL compounds that, at the same time, give origin to thermoresponsive solutions when dissolved in water, and are made ions that are relatively simple, chemically stable, moderately- or non-toxic and easy to keep under confinement, opening the way to a host of applications. The earliest applications, and those that up to now are the most relevant in practice, are in chemical processing, and, in particular, concern extraction, separation and purification technologies, suitable for large-scale applications, such as, for example, the extraction of rare earth ions from water or of contaminants from hydrocarbons, but also suitable for niche applications in biochemistry and in biotechnology, drug preparation and delivery. Examples of this type of lab-scale applications are represented by the extraction, purification and preservation of proteins and genetic material in their native form, the purification of drugs, and their release in response to a change of temperature from room to body temperature. Another major group of applications in chemical processing concerns catalysis, in which the phase separation is used first to perform the catalytic reaction in the homogeneous phase, with optimal contact of all components, and the successive phase separation under a temperature change is exploited to separate the products from the solvent, and to recover the catalyst. In extraction, separation, purification, and catalysis, thermo-responsiveness is primarily used to favour the kinetics of the entire process, bypassing diffusion-limited steps in the separation of solvent and target products in the final stages of these processes.

Applications that are already developed and, to some extent, deployed, at least at the laboratory scales, are only briefly covered in the present paper, mainly because they are the topic of recent and comprehensive reviews. We selected instead to discuss in some detail water, energy, and environmental applications, which are still in very preliminary stages of development (FO desalination using thermoresponsive IL/water draw solutions), or still at the level of speculation, such as IL/water solutions for permanently storing low grade heat, and water harvesting from the atmosphere. Of these, FO desalination based on thermoresponsive IL/water draw solutions already has a well established factual basis, and represents a technology that only needs further materials science and engineering developments to overcome an industrial and economic handicap to break into the open and gain a foothold in the expanding market for fresh water. At the very least, it is likely to play a role in the desalination of non-seawater resources, such as, for instance, brine from oil and gas wells, having high salinity and a variety of contaminants.

The expected practical and conceptual development of thermoresponsive IL/water (and, more in general, IL/solvent) systems will depend on the extension to this research area of old (combinatorial chemistry) and new (machine learning) tools able to explore a wider portion of the vast number of IL/solvent systems. The aim of new research will be to better understand the role of cations, and to explore a variety of different or intermediate systems, such as IL/polymers, poly-ILs in water and in organic solvents, ILs in hydrogels, nanoparticles in water and ILs, deep eutectic systems, as well as systems with a higher number of components. Another open research direction concerns multi-responsive systems, reacting to the combination of stimuli that involve temperature, but also light, pH, magnetic fields, addition of gases or chemical contaminants. Both research directions dramatically increase the number and variety of systems and properties of interest. Developments along these lines, fortunately or unfortunately, will make the exploration of thermoresponsive, IL-related solutions an endless effort, whose impact on applications can also be very extensive and profound.

Finally, any discussion of future applications of ILs cannot neglect considerations on the cost of these compounds. One cannot be too specific because the cost of ILs depends very sensitively on which IL is considered, and the cost will go down in the case of bulk production, but it is likely that it will remain high compared to that of traditional organic solvents [197]. It might be useful to remark, however, which applications of thermoresponsive IL/solvent solutions rely precisely on the ability to separate easily and virtually completely the IL following demixing. Therefore, the IL component will need to be replenish only slightly after each application cycle, reducing the impact of the high cost of ILs. In forward osmosis desalination, which could require large amounts of solute, the recovery of the IL can be substantially better than the ratio (10 wt% of IL remaining in the water-rich phase) resulting from the spontaneous phase separation when supplemented by a second stage of recovery, which is needed in any case to obtain fresh water of drinkable quality.

## Data Availability

Not applicable.
